# Synthesis of acylhydrazino-peptomers, a new class of peptidomimetics, by consecutive Ugi and hydrazino-Ugi reactions

**DOI:** 10.3762/bjoc.12.285

**Published:** 2016-12-27

**Authors:** Angélica de Fátima S Barreto, Veronica Alves dos Santos, Carlos Kleber Z Andrade

**Affiliations:** 1 Laboratório de Química Metodológica e Orgânica Sintética, Instituto de Química, Universidade de Brasília, CP 4478, 70910-970 Brasília-DF, Brazil

**Keywords:** acylhydrazino-peptomers, consecutive Ugi reactions, peptide-peptoid hybrid, peptomer

## Abstract

Herein we describe a versatile approach for the synthesis of acylhydrazino-peptomers, a new class of peptidomimetics. The key idea in this approach is based on a simple route using a one-pot hydrazino-Ugi four-component reaction followed by a hydrazinolysis or hydrolysis reaction and subsequent hydrazino-Ugi reaction or classical Ugi reaction for the construction of acyclic acylhydrazino-peptomers. The consecutive multicomponent reactions produced a variety of acylhydrazino-peptomers in moderate to excellent yields (47–90%). These compounds are multifunctional intermediates that can be further functionalized to obtain new peptidomimetics with potential biological activity.

## Introduction

**Figure 1:**
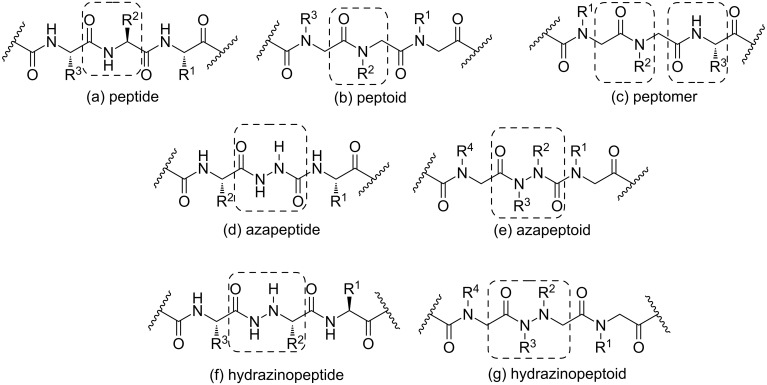
Structural features of (a) peptide, (b) peptoid, (c) peptomer, (d) azapeptide, (e) azapeptoid, (f) hydrazinopeptide and (g) hydrazinopeptoid.

**Figure 2:**
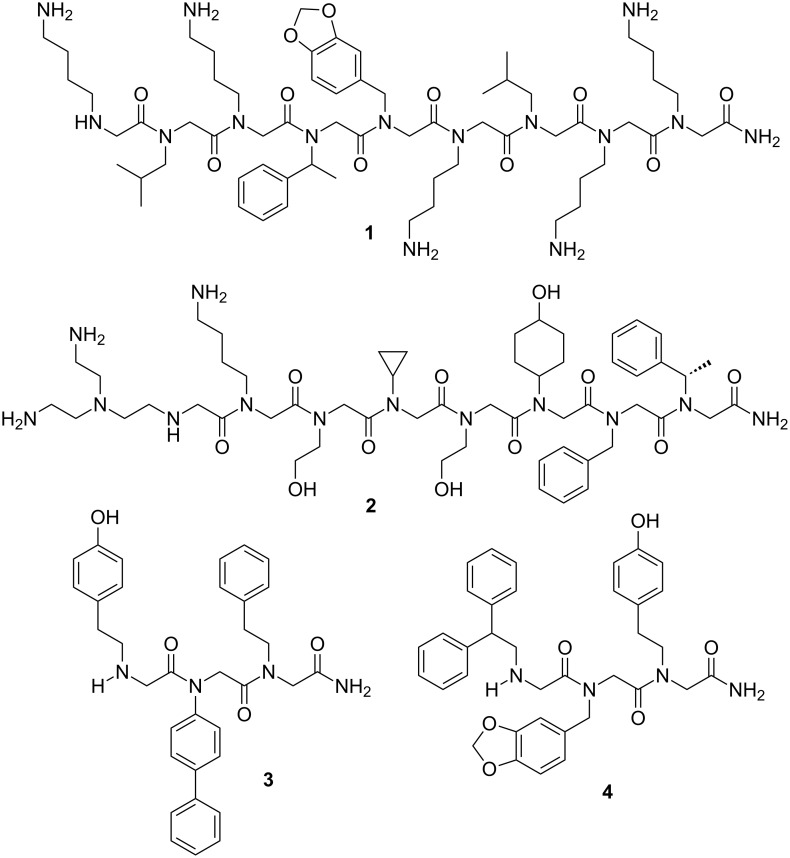
Some biologically active peptoids.

In the last decades, increasing efforts have been extensively carried out to improve the pharmacological properties of natural peptides by structural modification of the amino acids [1–8]. These modifications allowed the obtention of molecules that mimic the properties of peptides (peptidomimetics) but usually exhibit greater proteolytic stability, increased cellular permeabilities and avoid stereochemical constraints. Figure 1 represents some of the most important classes of peptidomimetics so far obtained and highlights the differences among them. Of these, peptoids (oligomers of *N*-substituted glycine residues) [9–12] are the most common and may have interesting biological activities. For instance, peptoid **1** is a target for cancer therapeutics for being an antagonist of the vascular endothelial growth factor receptor 2 [13]; peptoid **2** is a ligand of the protooncogene Crk [14]; and peptoids **3** and **4** showed a high affinity for the α1-adrenergic and μ-specific opiate receptors [15], respectively (Figure 2).

Unlike peptides, in peptoids the side chain of the C^α^ is bound to the nitrogen atom. Due to the consequent lack of the polar N–H bonds, their lipophilicity is increased, which may result in improved membrane permeability [16–17]. Furthermore, peptoids have also found utility in supra- and macromolecular engineering [18] and polymer chemistry [19]. Nevertheless, the lack of constraints in peptoids may result in a lower affinity for macromolecular targets, which limits their utility [20]. This finding has motivated the search for efficient strategies to introduce structural constraints into peptoid structures, which has contributed to increase the plethora of currently available peptidomimetics.

Azapeptides [21–23] are peptide analogs in which the C^α^ atom is substituted by a nitrogen atom in one or more amino acids (Figure 1) and have been known since 1970 [24]. The introduction of a semicarbazide moiety has an enormous effect on the physical properties of a peptide as well as on its structural characteristics. The semicarbazide constraints tend to facilitate interactions with protein receptors due to turn geometry within the azapeptide. As a result, their stability over enzymes and chemical degradation may be enhanced compared to a natural peptide. Indeed, these compounds have shown to be a useful class of peptidomimetics with interesting biological activities [21–23], including antiviral [25–26] and cysteine protease inhibition [27–30].

**Figure 3:**
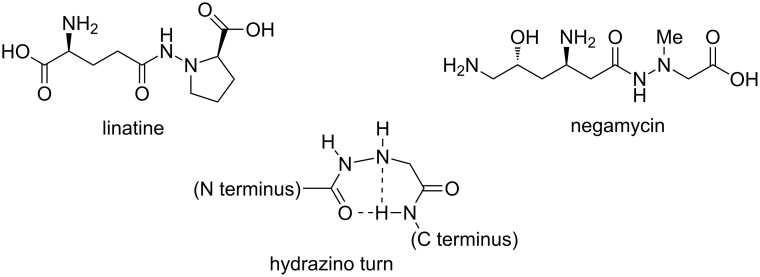
Biologically active hydrazinopeptides and representation of the hydrazino turn.

Hydrazinopeptides [31–36] (peptide analogs in which one of the CONH links is replaced by a hydrazido fragment CONHNH, Figure 1) represent another class of peptidomimetics with promising conformational and biological activities, such as protease inhibition [37] and antimicrobial activity [38]. There are some natural peptides that contain such an α-hydrazino acid moiety, e.g., the vitamin B6 antagonist linatine and the antibiotic negamycin (Figure 3). In the early 1970s, the first attempts to peptide modifications by hydrazino acids generated bioactive pseudopeptides [39]. Analogously to azapeptides, these compounds possess a conformational constraint (hydrazino turn) due to the presence of intramolecular H-bonding interactions (Figure 3), which is similar in nature to a natural peptide β-turn. As already pointed out, this can improve the proteolytic stability of the natural peptide while preserving its biological activity.

Other changes in peptoid structures have also been carried out to obtain the less common but also biologically active azapeptoids [40], hydrazino-azapeptoids [41–42], retro hydrazino-azapeptoids [43] and peptoid-azapeptoid hybrids [39]. Recently, Seo et al. [44] reported the synthesis of a library of peptide-peptoid hybrid (termed peptomers by Ostergaard and Holm [45]) prodrugs that can be selectively activated by prostate cancer cells.

**Figure 4:**
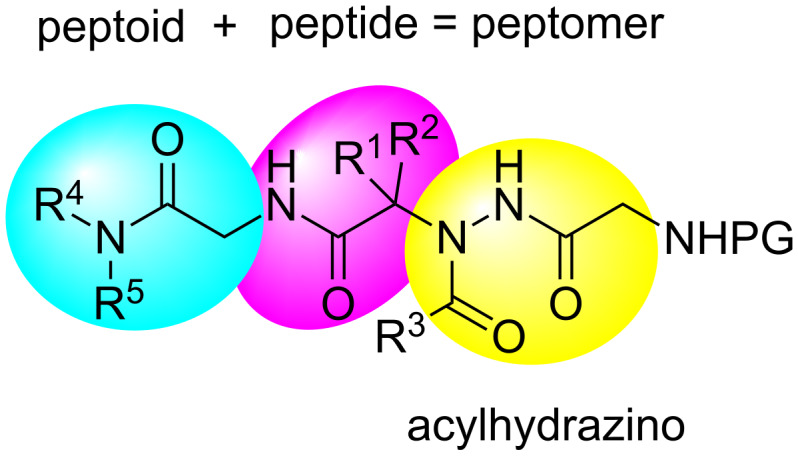
General structure of the acylhydrazino-peptomers synthesized in this study.

Peptidomimetics can be conveniently synthesized using the so called "submonomer approach" either in solution [41] or in solid-phase [46–47]. However, some disadvantages have been reported for long or difficult sequences [48]. It is therefore important to have alternative methods for the fast and easy construction of such important compounds. In this sense, the Ugi four-component reaction (U-4CR) has proven to be a robust and versatile method for the synthesis of peptoids and peptide-peptoid hybrids (peptomers) [49–53]. This reaction has also been combined with other protocols for the synthesis of bioactive peptides [54–55] and hydrazinopeptide motifs [33–34]. In continuing our research on the synthesis of peptoids [53,56–57], herein we describe the synthesis of a new class of peptidomimetics, which we have called acylhydrazino-peptomers (Figure 4), by analogy with the existing classes of peptidomimetics shown in Figure 1, using consecutive Ugi and hydrazino-Ugi reactions, respectively. These compounds comprise both a peptoid and a peptide moiety (hence a peptomer) along with an acylhydrazino portion. The use of a consecutive Ugi reaction to access peptidomimetics has proven very useful [56–62]. It is important to point out that these molecules cannot be obtained directly via the “submonomer approach”.

## Results and Discussion

**Scheme 1:**
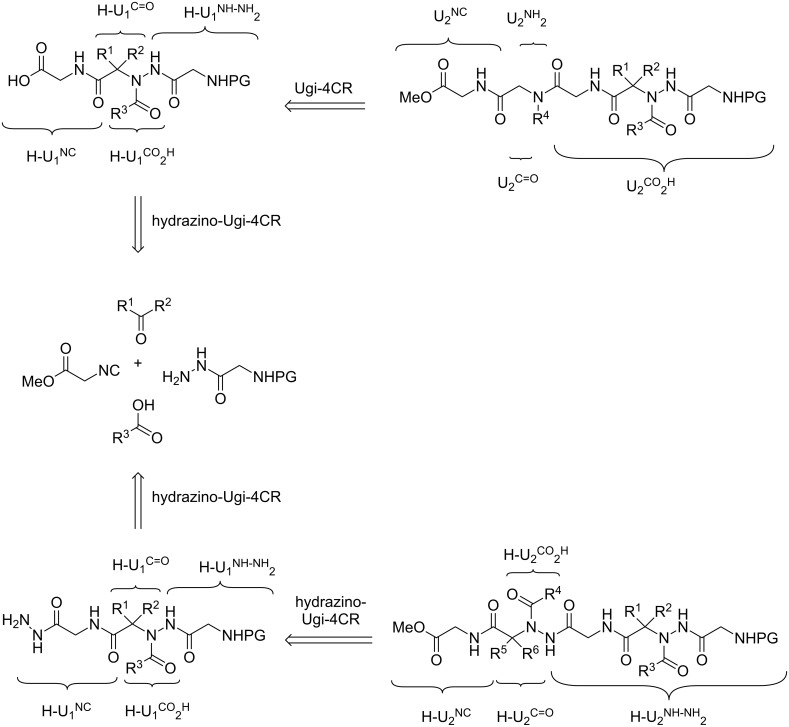
Retrosynthetic analysis.

Our approach involves the use of two multicomponent reactions (Scheme 1): the hydrazino-Ugi four-component reaction (HU-4CR) and the classical Ugi reaction (U-4CR). The strategy was based on the formation of an acylhydrazino-peptomer via an initial hydrazino-Ugi reaction followed by a hydrazinolysis reaction (or ester hydrolysis) and a subsequent hydrazino-Ugi reaction (or a classical Ugi reaction).

**Scheme 2:**
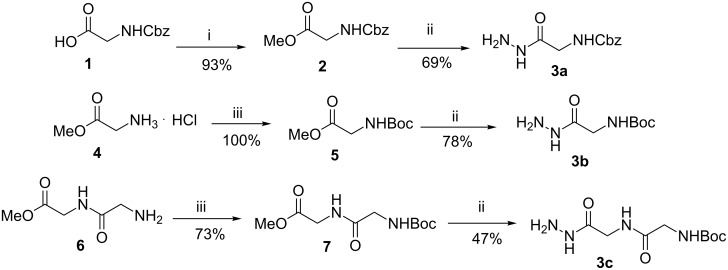
Synthesis of hydrazides **3a–c**. Reagents and conditions: (i) CH_3_I, NaHCO_3_, DMF, rt, 46 h; (ii) N_2_H_4_^.^H_2_O, EtOH, reflux, 2–3 h; (iii) (Boc)_2_O, NaOH, dioxane/H_2_O, overnight.

The hydrazides **3a–c** used in the first MCR were prepared by the reaction of glycine-derived esters **2**, **5** and **7** with hydrazine monohydrate (hydrazinolysis), following a known procedure [63–64] (Scheme 2).

**Scheme 3:**
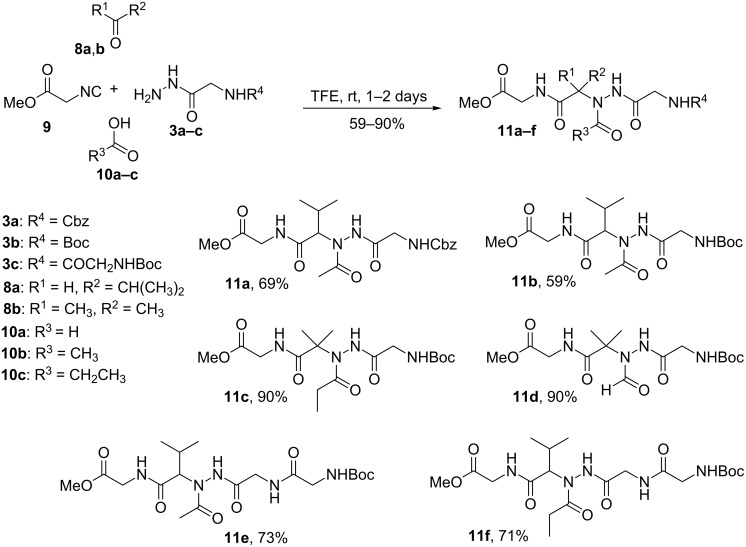
Synthesis of acylhydrazino-peptomers **11a–f**.

The obtained hydrazides were then reacted with isobutyraldehyde **8a**/acetone **8b**, carboxylic acids **10a–c** (formic, acetic or propionic acid) and ethyl isocyanoacetate **9** (Scheme 3). The reactions were conducted at room temperature in trifluoroethanol (TFE) for 1–2 days to yield the acylhydrazino-peptomers **11a–f** in moderate to good yields (59–90%). Aliphatic aldehydes, ketones and carboxylic acids succeeded in the hydrazino-Ugi reaction, except for paraformaldehyde, fatty acids and aromatic substrates, which led to formation of a complex mixture. These results limited the obtention of a greater variety of acylhydrazino-peptomers by this method. The choice of TFE as the solvent for the hydrazino-Ugi reaction was important because the same reaction carried out in methanol, often the solvent of choice for Ugi reactions, provided the formation of a complex mixture. This fact has already been reported [34].

**Scheme 4:**
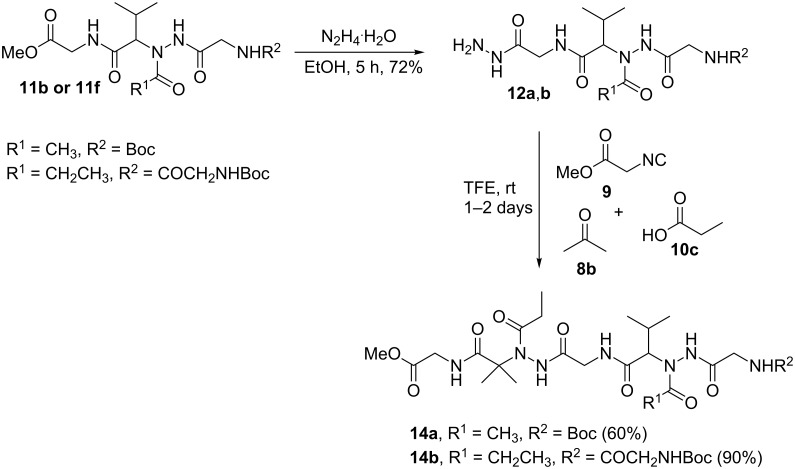
Synthesis of acylhydrazino-peptomers **14a**,**b**.

**Scheme 5:**
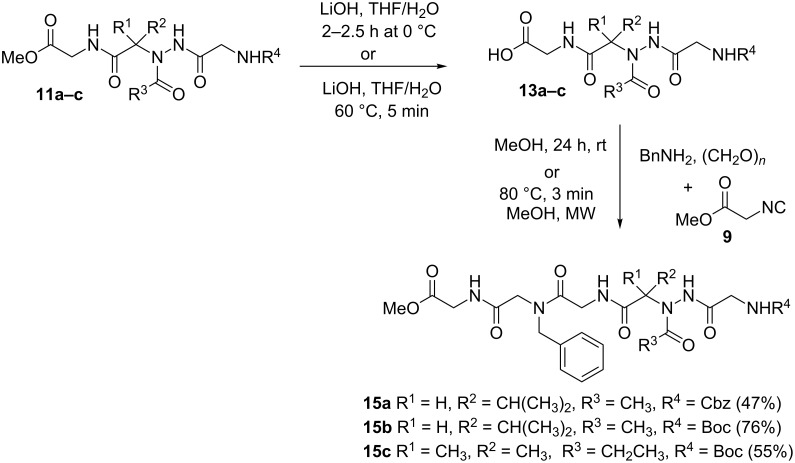
Synthesis of acylhydrazino-peptomers **15a–c**.

To further functionalize the acylhydrazino-peptomers structures, a second Ugi reaction was carried out. Hence, some acylhydrazino-peptomers were subjected to hydrazinolysis reaction or ester hydrolysis to give the corresponding hydrazides **12a**,**b** (Scheme 4) or acids **13a–c** (Scheme 5), respectively, which were used in the following step (hydrazino-Ugi reaction or classical Ugi reaction) to yield the corresponding acylhydrazino-peptomers **14a,b** or **15a–c** in moderate to good yields (47–90%). All compounds were fully characterized by ^1^H and ^13^C NMR and HRMS giving data consistent with the proposed structures.

Functionalization on both termini of compounds **11**, **14** and **15** (ester hydrolysis and/or Boc deprotection) allows subsequent Ugi or hydrazino-Ugi reactions to further elongate the peptomers main chain.

## Conclusion

In summary, we have developed a concise protocol for the synthesis of functionalized acylhydrazino-peptomers by consecutive Ugi reactions. The general route allows an easy access to highly functionalized peptomers in good yields and a reduced number of steps. This method may also be employed to obtain new classes of peptidomimetics with potential biological activity.

## Supporting Information

File 1 Detailed experimental procedures, NMR and mass spectra of all compounds.
