# Supplementary material for: Synthesis of acylhydrazino-peptomers, a new class of peptidomimetics, by consecutive Ugi and hydrazino-Ugi reactions
Source: Beilstein J Org Chem. 2016 Dec 27;12:2865–72. doi: 10.3762/bjoc.12.285 (PMC5238544; doi:10.3762/bjoc.12.285)
Supplement: File 1 — Detailed experimental procedures, NMR and mass spectra of all compounds. [file Beilstein_J_Org_Chem-12-2865-s001.pdf]

Supporting Information

for

Synthesis of acylhydrazino-peptomers, a new class of peptidomimetics, by consecutive Ugi and hydrazino-Ugi reactions

Angélica de Fátima S. Barreto,* Veronica Alves dos Santos and Carlos Kleber Z. Andrade*

Address: Laboratório de Química Metodológica e Orgânica Sintética, Instituto de Química, Universidade de Brasília, CP 4478, 70910-970 Brasília-DF, Brazil.

Email: Carlos Kleber Z. Andrade* - ckleber@unb.br

*Corresponding author

Detailed experimental procedures, NMR and mass spectra of all compounds

General information S2
General procedure for the preparation of hydrazides S2
General procedure for the preparation of *Boc*-protected amido acids S3
References S14
Spectra of compounds S15–S57

Experimental

General information

NMR spectra were recorded on a Bruker Ascend instrument using a 5-mm internal diameter probe operating at 600 MHz for ^{1}H and at 150 MHz for ^{13}C or in a Varian Mercury Plus 300 spectrometer at 300 MHz for ^{1}H and 75.46 MHz for ^{13}C both in the presence of TMS as internal standard. High resolution ESI(+)-MS analyses were carried out on a triple TOF 5600+ (AB Sciex) with internal calibration and direct solution (1 ppm) infusion. Reactions under microwave were performed on a CEM Co. Discover microwave reactor using sealed vessels, dynamic program, and temperature detection by internal fiber optic probe and media stirring. TLC plates were revealed by treatment with a 10% solution of phosphomolybdic acid in ethanol, followed by heating. Melting points were recorded on a Marconi melting point and are uncorrected. Commercially available reagents and solvents were analytical grade or were purified by standard procedures prior to use. Compounds were analyzed by ^{1}H NMR, ^{13}C NMR and high resolution ESI mass spectra giving data consistent with the proposed structures.

General procedure for the preparation of hydrazides [1]

The ester (10.0 mmol) was added in small portions to a solution of hydrazine hydrate (40.0 mmol) in 3.0 mL of ethanol. After refluxing for 3–6.5 h, the residue was concentrated in vacuum and purified by column chromatography.

General procedure for the preparation of *Boc*-protected amino acids [2]

The amino acid (10.0 mmol) was dissolved in dioxane/water (2:1, 30 mL), which was made alkaline with NaOH (1 M, 10 mL) and cooled in an ice-bath, and $(Boc)_2O$ (3.27 g, 15.0 mmol) and $NaHCO_3$ (0.84 g, 10.0 mmol) were added. The reaction mixture was stirred overnight at room temperature and was then evaporated to half the volume. The residue was diluted with EtOAc (40 mL), cooled in an ice-bath and acidified to pH = 2.5–3.0 with $KHSO_4$ (1 M). The layers were separated, the aqueous fraction was extracted with EtOAc (2 × 20 mL), and the combined organic extracts were washed with water, dried and evaporated to yield the respective *Boc*-Amino acid, which was used in the next step without further purification.

***Cbz*-glycine hydrazide (3a)** [3]

To a stirred solution of *Cbz*-glycine (**1**, 2.56 mmol, 0.54 g) in DMF (3.0 mL) was added sodium bicarbonate (3.38 mmol, 0,28 g) followed by methyl iodide (9.64 mmol, 0.60 mL). The mixture was stirred under nitrogen atmosphere for 46 h at room temperature. After this time, 30 mL of ethyl acetate was added and the mixture was washed with distilled water (three times). The organic phase was separated, dried with sodium sulfate and concentrated to yield product **2** (2.38 mmol, 0.53 g, 93% yield), which was used without further purification. Compound **3a** was prepared following the general procedure for the preparation of hydrazide (refluxing for 3 h) using product **2** (4.93 mmol, 1.10 g), hydrazine hydrate (17.2 mmol, 0.86 g) and 1.4 mL of ethanol. Purification by column chromatography (CH_2Cl_2 → 10% MeOH/CH_2Cl_2) furnished **3a** in 69% yield (0.76 g, 3.42 mmol) as a white solid. R_f (CH_2Cl_2/MeOH 15%) = 0.51. m.p = 112-

114 ºC. ^{1}H NMR (300 MHz, CD_3OD) δ 7.37-7.27 (m, 5H), 5.09 (s, 2H), 3.76 (s, 2H). ^{13}C NMR (75.46 MHz, $CDCl_3$) δ 171.4, 158.9, 138.0, 129.4, 129.0, 128.9, 67.8, 43.6.

***Boc*-glycine hydrazide (3b)** [4]

Compound **5** was prepared following the general procedure for the preparation of *Boc*-amino acids using glycine methyl ester hydrochloride (1.25 g, 10.0 mmol) in quantitative yields as a colorless oil, which was used without further purification. Compound **3b** was then prepared following the general procedure for the preparation of hydrazide (refluxing for 3 h) using ester **5** (9.90 mmol, 1.87 g), hydrazine hydrate (39.6 mmol, 1.98 g) and 3.0 mL of ethanol. Purification by column chromatography (CH_2Cl_2 → 10% MeOH/CH_2Cl_2) furnished *Boc*-glycine hydrazide (**3b**) in 78% yield (1.45 g, 7.67 mmol) as a white solid. R_f (CH_2Cl_2/MeOH 15%) = 0.43. m.p = 105-107 ºC. ^{1}H NMR (300 MHz, CD_3OD) δ 3.68 (s, 2H), 1.44 (s, 9H). ^{13}C NMR (75.46 MHz, CD_3OD) δ 171.9, 158.6, 80.9, 43.5, 28.8.

Hydrazide 3c

Compound **7** was prepared in 73% yield as a viscous colorless oil following the general procedure for the preparation of *Boc*-Amino acids using amine **6** (1.46 g, 10.0 mmol) and was used without further purification. Compound **3c** was then prepared following the general procedure for the preparation of hydrazide (refluxing for 3 h) using ester **7** (4.35 mmol, 1.07 g), hydrazine hydrate (17.4 mmol, 0.87 g) and 2.3 mL of ethanol. Purification by column chromatography (CH_2Cl_2 → 15% MeOH/CH_2Cl_2) furnished hydrazide **3c** in 47% yield (0.51 g,

2.05 mmol) as a white solid. R_f (CH_2Cl_2/MeOH 15%) = 0.24. m.p = 44-46 ºC. ^1H NMR (300 MHz, CD_3OD) δ 3.87 (s, 2H), 3.73 (s, 2H), 1.46 (s, 9H). ^{13}C NMR (75.46 MHz, $CDCl_3$) δ 173.3, 170.9, 158.8, 81.0, 44.9, 42.4, 28.8. HRMS (ESI) *m/z*: calcd. for $[M+Na]^+$ $C_9H_{18}N_4O_4Na$: 269.1226; found: 269.1217.

Compound 11a

To a stirred solution of *Cbz*-glycine hydrazide (**3a**, 2.13 mmol, 0.476 g) in methanol (4.0 mL) was added isobutyraldehyde (**8a**, 2.13 mmol, 0.19 mL) and stirring was continued for 0.5 h at room temperature. After the solvent was evaporated and the imine was dissolved in trifluoroethanol (4.5 mL), acetic acid (**10b**, 2.13 mmol, 0.122 mL) and methyl isocyanoacetate (**9**, 2.13 mmol, 0.19 mL) were added. After stirring for 18 h at room temperature, the solution was concentrated in vacuum and the residue was purified by column chromatography (CH_2Cl_2 → 2% MeOH/CH_2Cl_2) to yield ester **11a** (1.47 mmol, 0.64 g, 69% yield) as a beige solid. R_f (CH_2Cl_2/MeOH 10%) = 0.54. m.p = 103-105 ºC. ^1H NMR (600 MHz, $CDCl_3$, presence of rotamers) δ 9.09-8.89 (2 br s, 1H), 7.35-7.30 (m, 5H), 7.13 (br s, 1H), 5.82 and 5.58 (2 br s, 1H), 5.13 (s, 2H), 4.72 (d, J = 9.5 Hz, 1H), 4.06-3.84 (m, 4H), 3.72 (s, 3H), 2.24-2.12 (m, 1H), 2.07 (s, 3H), 1.02-0.91 (m, 6H).^{13}C NMR (150 MHz, $CDCl_3$, presence of rotamers): δ 174.2; 171.2; 170.3; 168.3; 156.6; 136.0; 128.5; 128.0; 67.3; 64.3; 52.4; 43.3; 40.9; 27.2; 20.9; 19.8; 19.3. HRMS (ESI) *m/z*: calcd. for $[M+Na]^+$ $C_{20}H_{28}N_4O_7Na$: 459.1856; found: 459.1848.

Compound 11b

To a stirred solution of *Boc*-glycine hydrazide (**3b**, 0.50 mmol, 0.094 g) in methanol (1.0 mL) was added isobutyraldehyde (**8a**, 0.50 mmol, 0.45 mL) and stirring was continued for 2 h at room temperature. After the solvent was evaporated and the imine was dissolved in trifluoroethanol (2.0 mL), acetic acid (**10b**, 0.25 mmol, 0.014 mL) and methyl isocyanoacetate (**9**, 0.25 mmol, 0.023 mL) were added. After stirring for 43 h at room temperature, the solution was concentrated in vacuum and the residue was purified by column chromatography ($CH_2Cl_2 \rightarrow$ 2% MeOH/CH_2Cl_2) to yield ester **11b** (0.293 mmol, 0.118 g, 59%) as a white solid. R_f (CH_2Cl_2/MeOH 10%) = 0.51. m.p. = 160-162 ºC. ^{1}H NMR (300 MHz, $CDCl_3$, presence of rotamers) δ 8.99 (br s, 1H), 6.83 (br s, 1H), 5.16 (br s, 1H), 4.64 (br s, 1H), 3.97 (s, 2H), 3.82 (s, 2H), 3.73 (s, 3H), 2.27-2.14 (m, 1H), 2.07 (s, 3H), 1.42 (s, 9H), 1.02 (s, 3H), 0.95 (d, *J* = 6.6 Hz, 3H). ^{13}C NMR (150 MHz, DMSO-d_6, presence of rotamers): δ 172.3, 170.8, 169.9, 169.1, 155.8, 78.3, 63.4, 51.6, 42.4, 40.5, 28.1, 26.9, 20.6, 19.1, 18.7. HRMS (ESI) *m/z*: calcd. for $[M+Na]^+$ $C_{19}H_{27}N_3O_6Na$: 425.2012; found: 425.2012.

Compound 11c

To a stirred solution of hydrazine **3b** (1.59 mmol, 0.30 g) in trifluoroethanol (2.0 mL) was added ketone **8b** (3.18 mmol, 0.23 mL) and stirring was continued for 2 h at room temperature. Then were added sodium sulfate (0.30 g), propanoic acid (**10c**, 0.79 mmol, 0.059 mL) and methyl isocyanoacetate (**9**, 0.79 mmol, 0.072 mL). After stirring for 40 h at room temperature, the solution was concentrated in vacuum and the residue was purified by column

chromatography ($CH_2Cl_2 \rightarrow$ 4% MeOH/CH_2Cl_2) to yield ester **11c** (0.714 mmol, 0.287 g, 90%) as a viscous light yellow oil. R_f (CH_2Cl_2/MeOH 10%) = 0.47. ^{1}H NMR (600 MHz, $CDCl_3$, presence of rotamers) δ 9.65 (br s, 1H), 8.73 (br s, 1H), 5.70 (br s, 1H), 4.11 (dd, *J* = 5.9 and 18.0 Hz, 1H), 3.97-3.96 (m, 2H), 3.92 (dd, *J* = 5.1 and 18.0 Hz, 1H), 3.71 (s, 3H), 2.42-2.36 and 2.25-2.19 (2m, 2H), 1.45 (s, 12H), 1.36 (s, 3H), 0.99 (t, *J* = 7.3 Hz, 3H). ^{13}C NMR (150 MHz, $CDCl_3$, presence of rotamers): δ 175.6, 175.1, 172.6, 170.3, 156.2, 80.4, 64.5, 52.1, 42.9, 41.6, 28.2, 26.2, 24.5, 22.2, 8.3. HRMS (ESI) *m/z*: calcd. for $[M+Na]^+$ $C_{17}H_{30}N_4O_7Na$: 425.2012; found: 425.2013.

Compound 11d

To a stirred solution of hydrazine **3b** (0.50 mmol, 0,094 g) in trifluoroethanol (1.0 mL) was added ketone **8b** (1.0 mmol, 0.073 mL) and stirring was continued for 1 h at room temperature. Then were added sodium sulfate (0.50 g), formic acid (**10a**, 0.25 mmol, 0.009 mL) and methyl isocyanoacetate (**9**, 0.25 mmol, 0.023 mL). After stirring for 24 h at room temperature, the solution was filtrated, concentrated in vacuum and the residue was purified by column chromatography ($CH_2Cl_2 \rightarrow$ 4% MeOH/CH_2Cl_2) to yield ester **11d** (0.25 mmol, 0.084 g, 90%) as a viscous light yellow oil. R_f (CH_2Cl_2/MeOH 10%) = 0.48. ^{1}H NMR (600 MHz, $CDCl_3$, presence of rotamers) δ 9.50 (br s, 1H), 8.69 and 8.59 (2 br s, 1H), 8.29 and 8.00 (2s, 1H), 5.52 (br s, 1H), 4.03-3.96 (m, 4H), 3.73 (s, 3H), 1.58 (s, 3H), 1.46-1.44 (m, 12H). ^{13}C NMR (150 MHz, $CDCl_3$, presence of rotamers): δ 174.4, 170.4, 164.2, 161.4, 156.1, 80.4, 63.9, 52.1, 42.6, 41.5, 28.2, 25.5, 23.6. HRMS (ESI) *m/z*: calcd. for $[M+Na]^+$ $C_{15}H_{26}N_4O_7Na$: 397.1699; found: 397.1703.

Compound 11e

To a stirred solution of hydrazine **3c** (2.13 mmol, 0,524 g) in methanol (4.0 mL) was added isobutyraldehyde (**8a**, 2.13 mmol, 0.19 mL) and stirring was continued for 1 h at room temperature. After the solvent was evaporated and the imine was dissolved in TFE (4.0 mL), were added acetic acid (**10b**, 2.13 mmol, 0.12 mL) and methyl isocyanoacetate (**9**, 2.13 mmol, 0.19 mL). After stirring for 48 h at room temperature, the solution was concentrated in vacuum and the residue was purified by column chromatography (CH_2Cl_2 → 2% MeOH/CH_2Cl_2) to yield the ester **11e** (1.55 mmol, 0.710 g, 73%) as a yellow solid. R_f (CH_2Cl_2/MeOH 10%) = 0.38. m.p = 57-59 ºC. ^{1}H NMR (600 MHz, $CDCl_3$, presence of rotamers) δ 9.32 and 9.25 (2s, 1H), 7.53 and 7.49 (2s, 1H), 5.63 and 5.53 (2s, 1H), 4.61 (d, J = 9.2 Hz, 1H), 4.00 and 3.87 (2 br s, 6H), 3.75 (s, 3H), 2.25-2.15 (m, 1H), 2.08 (s, 3H), 1.44 (s, 9H), 1.10-0.97 (m, 6H). ^{13}C NMR (150 MHz, $CDCl_3$, presence of rotamers): δ 174.1, 171.0, 170.4, 169.7, 168.3, 156.5, 80.5, 65.2, 52.4, 44.2, 41.9, 41.0, 27.2, 20.9, 19.8, 19.4, 19.2. HRMS (ESI) *m/z*: calcd. for $[M+Na]^+$ $C_{19}H_{33}N_5O_8Na$: 482.2227; found: 482.2228.

Compound 11f

To a stirred solution of hydrazine **3c** (1.62 mmol, 0,398 g) in methanol (3.0 mL) was added isobutyraldehyde (**8a**, 1.62 mmol, 0.15 mL) and stirring was continued for 1 h at room temperature. After the solvent was evaporated and the imine was dissolved in TFE (3.0 mL), were added propanoic acid (**10c**, 1.62 mmol, 0.12 mL) and methyl isocyanoacetate (**9**, 1.62 mmol, 0.15 mL). After stirring for 51 h at room temperature, the solution was concentrated in vacuum

and the residue was purified by column chromatography (CH_2Cl_2 → 4% MeOH/CH_2Cl_2) to yield ester **11f** (1.15 mmol, 0.543 g, 71%) as a light yellow solid. R_f (CH_2Cl_2/MeOH 10%) = 0.46. m.p = 53-55 ºC. ^{1}H NMR (600 MHz, $CDCl_3$, presence of rotamers) δ 9.27 and 9.20 (2 br s, 1H), 7.58 (br s, 1H), 7.33 (br s, 1H), 5.68 and 5.57 (2 br, 1H), 4.63 (s, 1H), 4.20-3.78 (m, 6H), 3.74 (s, 3H), 2.47-2.31 (m, 2H), 2.27-2.17 and 2.14-2.05 (2m, 1H), 1.44 (s, 9H), 1.14-0.91 (m, 9H). ^{13}C NMR (150 MHz, $CDCl_3$, presence of rotamers): δ 177.1, 171.3, 170.4, 169.6, 168.4, 156.4, 80.5, 65.6, 52.4, 44.2, 41.9, 41.0, 28.3, 27.3, 25.7, 20.0, 19.5, 8.6. HRMS (ESI) *m/z*: calcd. for $[M+Na]^+$ $C_{20}H_{35}N_5O_8Na$: 496.2383; found: 496.2379.

Hydrazide 12a

Compound **12a** was prepared following the general procedure for the preparation of hydrazides (refluxing 5 h) using ester **11b** (0.71 mmol, 0.286 g), hydrazine hydrate (2.84 mmol, 0,14 mL) and 3.3 mL of ethanol. Purification by column chromatography (CH_2Cl_2 → 15% MeOH/CH_2Cl_2) furnished hydrazide **12a** in 72% yield (0.51 mmol, 0.205 g) as a white solid. R_f (CH_2Cl_2/MeOH 15%) = 0.29. m.p = 59-61 ºC. ^{1}H NMR (600 MHz, CD_3OD, presence of rotamers) δ 4.63 (d, *J* = 9.5 Hz, 1H), 3.96-3.68 (m, 4H), 2.24-2.12 (m, 1H), 1.45 (s, 9H), 1.01-0.89 (m, 6H). ^{13}C NMR (150 MHz, CD_3OD, presence of rotamers) δ 175.9, 173.1, 171.7, 170.6, 158.6, 80.9, 64.9, 43.4, 42.9, 28.8, 27.9, 20.9, 20.5, 19.5. HRMS (ESI) *m/z*: calcd. for $[M+Na]^+$ $C_{16}H_{30}N_6O_6Na$: 425.2125; found: 425.2124.

Hydrazide 12b

Compound **12b** was prepared following the general procedure for the preparation of hydrazides (refluxing 5 h) using ester **11f** (0.890 mmol, 0.421 g), hydrazine hydrate (3.56 mmol, 0.17 mL) and 4.0 mL of ethanol. Purification by column chromatography (CH_2Cl_2 → 15% MeOH/CH_2Cl_2) furnished hydrazide **12b** in 72% yield (0.64 mmol, 0.304 g) as a white solid. R_f (CH_2Cl_2/MeOH 15%) = 0.31. m.p = 101-103 ºC. ^{1}H NMR (600 MHz, CD_3OD) δ 4.67 (d, *J* = 9.5 Hz, 1H), 4.04-3.66 (m, 6H), 2.46-2.40 (m, 1H), 2.32-2.13 (m, 2H), 1.45 (s, 9H), 1.07 (t, *J* = 7.5 Hz, 3H), 1.02-0.91 (m, 6H). ^{13}C NMR (150 MHz, CD_3OD) δ 178.6, 173.4, 172.1, 171.7, 170.5, 158.6, 80.7, 64.8, 44.7, 42.8, 42.2, 28.7, 27.8, 26.7, 20.3, 19.4, 9.4. HRMS (ESI) *m/z*: calcd. for $[M+H]^+$ $C_{19}H_{36}N_7O_7$: 474.2676; found: 474.2670.

Compound 14a

To a stirred solution of hydrazide **12a** (0.472 mmol, 0.190 g) in trifluoroethanol (2.0 mL) was added ketone **8b** (1.89 mmol, 0.14 mL) and stirring was continued for 1 h at room temperature. Then were added propanoic acid (**10c**, 0.472 mmol, 0.035 g) and methyl isocyanoacetate (**9**, 0.472 mmol, 0.043 mL). After stirring for 45 h at room temperature, the solution was concentrated in vacuum and the residue was purified by column chromatography (CH_2Cl_2 → 8% MeOH/CH_2Cl_2) to yield compound **14a** (0.281 mmol, 0.173 g, 60% yield) as a yellow solid. R_f (CH_2Cl_2/MeOH 10%) = 0.48. m.p = 118-120 ºC. ^{1}H NMR (600 MHz, CD_3OD) δ 4.64 (d, *J* = 9.5 Hz, 1H), 4.31-3.74 (m, 6H), 3.72 and 3.69 (2s, 3H), 2.43-2.32 (m, 1H), 2.26-2.13 (m, 2H), 2.07-2.02 (m, 3H), 1.46-1.31 (m, 15H), 1.03-0.88 (m, 9H). ^{13}C NMR (150 MHz, DMSO-*d_6*) δ 175.3, 173.8, 173.6,

172.1, 170.1, 169.2, 168.7, 155.8, 78.2, 63.5, 61.5, 51.6, 51.4, 48.7, 40.8, 28.0, 25.7, 24.0, 22.4, 20.6, 19.3, 18.8, 8.4. HRMS (ESI) *m/z*: calcd. for $[M+Na]^+$ $C_{26}H_{45}N_7O_{10}Na$: 638.3126; found: 638.3115.

Compound 14b

To a stirred solution of hydrazide **12b** (0.361 mmol, 0.171 g) in trifluoroethanol (2.0 mL) was added ketone **8b** (1.44 mmol, 0.11 mL) and stirring was continued for 3 h at room temperature. Then were added propanoic acid (**10c**, 0.180 mmol, 0.013 mL) and methyl isocyanoacetate (**9**, 0.180 mmol, 0.016 mL). After stirring for 40 h at room temperature, the solution was concentrated in vacuum and the residue was purified by column chromatography (CH_2Cl_2 → 8% MeOH/CH_2Cl_2) to yield compound **14b** (0.161 mmol, 0.076 g, 90% yield) as a yellow solid. R_f (CH_2Cl_2/MeOH 10%) = 0.39. m.p = 107-109 ºC. ^{1}H NMR (600 MHz, $CDCl_3$) δ 9.81 and 9.74 (2br s, 1H), 9.67 and 9.63 (2br s, 1H), 8.88 and 8.79 (2 br s, 1H), 8.58 and 8.26 (2 br s, 1H), 7.83 and 7.76 (2 br s, 1H), 5.88 and 5.81 (2 br s, 1H), 4.60 and 4.50 (s, 1H), 4.10-3.75 (m, 8H), 3.73 and 3.71 (2s, 3H), 2.49-2.39 (m, 1H), 2.35-2.18 (m, 4H), 1.46-1.34 (m, 15H), 1.06-0.92 (m, 12H). ^{13}C NMR (150 MHz, $CDCl_3$) δ 175.4; 175.2; 174.8; 171.4; 171.2; 170.8; 170.5; 170.1; 169.3; 80.9; 71.8; 64.5; 52.1; 49.4; 44.6; 42.2; 41.7; 41.2; 29.7; 28.3; 24.9; 22.0; 19.2; 14.1; 8.7. HRMS (ESI) *m/z*: calcd. for $[M+Na]^+$ $C_{29}H_{50}N_8O_{11}Na$: 709.3497; found: 709.3495.

Compound 15a

A sealed 10 mL glass tube containing a solution of ester **11a** (0.47 mmol, 0.205 g) in THF/H_2O (2:1, 7.5 mL) and LiOH (1.18 mmol, 0.028 g) at room

temperature was introduced in the cavity of a microwave reactor (CEM Co., Discover) and irradiated at 60 ºC for 5 min under magnetic stirring. The reaction mixture was then acidified with a 2 M solution of $NaHSO_4$ to pH 2 and extracted twice with ethyl acetate (2 × 30 mL). The organic phase was dried with sodium sulfate, filtered and concentrated to yield acid **13a** (0.44 mmol, 0.185 g, 94%), which was used without further purification. A mixture of acid **13a** (0.44 mmol, 0.185 g), methanol (1.5 mL), anhydrous sodium sulfate (0.20 g), paraformaldehyde (0.88 mmol, 0.0264 g), benzylamine (0.88 mmol, 0.094 g) and methyl isocyanoacetate (0.44 mmol, 0.040 mL) was introduced in the cavity of a microwave reactor (CEM Co., Discover) and irradiated at 80 ºC for 3 min under magnetic stirring. The product was filtered, concentrated in vacuum and purified by column chromatography (CH_2Cl_2 → 4% MeOH/CH_2Cl_2) to yield compound **15a** (0.207 mmol, 0.133 g, 47% yield) as a yellow solid. R_f (CH_2Cl_2/MeOH 10%) = 0.53. m.p = 87-89 ºC. ^{1}H NMR (600 MHz, $CDCl_3$) δ 9.90 and 9.57 (2 br s, 1H), 7.42 and 7.11 (m, 12H), 5.82 and 5.66 (2 br s, 2H), 5.10 (s, 2H), 4.82 (br s, 1H), 4.66-4.59 (m, 2H), 4.10-3.84 (m, 8H), 3.71 (s, 3H), 2.08-2.00 (m, 4H), 1.06-0.94 (m, 6H). ^{13}C NMR (150 MHz, $CDCl_3$) δ 171.0, 170.4, 170.3, 170.1, 168.5, 167.9, 156.7, 136.1, 134.7, 129.2, 128.9, 128.6, 128.3, 128.2, 126.8, 67.3, 64.4, 52.4, 51.8, 49.7, 43.5, 41.2, 41.0, 29.7, 27.1, 20.9, 19.6. HRMS (ESI) *m/z*: calcd. for $[M+Na]^+$ $C_{31}H_{40}N_6O_9Na$: 663.2754; found: 663.2755.

Compound 15b

To a solution of ester **11b** (1.96 mmol, 0.788 g) in THF/H_2O (2:1, 69 mL) was added LiOH (9.8 mmol, 0.235 g) at 0 ºC. The reaction was stirred for 2.5 h at 0

ºC, acidified with a 2 M solution of $NaHSO_4$ to pH 2 and extracted three times with ethyl acetate. The combined organic phases were dried with sodium sulfate and concentrated to yield the respective acid **13b** (1.96 mmol, 0.761 g, quantitative yield), which was used without further purification. A sealed 10 mL glass tube containing a mixture of acid **13b** (1.63 mmol, 0.633 g), methanol (2.0 mL), benzylamine (3.25 mmol, 0.348 g), anhydrous sodium sulfate (0.975 g), paraformaldehyde (3.25 mmol, 0.0975 g) and methyl isocyanoacetate (**9**, 1.63 mmol, 0.15 mL) was introduced in the cavity of a microwave reactor (CEM Co., Discover) and irradiated at 80 ºC for 3 min (ramp time: 100 s) under magnetic stirring. The residue was filtered, concentrated in vacuum and purified by column chromatography (CH_2Cl_2 → 4% MeOH/CH_2Cl_2) to yield compound **15b** (1.23 mmol, 0.748 g, 76% yield) as a yellow solid. R_f (CH_2Cl_2/MeOH 15%) = 0.53. m.p = 79-81 ºC. ^1H NMR (600 MHz, $CDCl_3$) δ 9.96 and 9.59 (2s, 1H), 7.37-7.22 (m, 5H), 4.84-4.76 (m, 1H), 4.71-4.60 (m, 2H), 4.25-3.78 (m, 8H), 3.74 (s, 3H), 2.16-1.95 (m, 4H), 1.43 (s, 9H), 1.11-1.02 (m, 2H), 0.95 (d, *J* = 6.2 Hz, 4H). ^{13}C NMR (150 MHz, $CDCl_3$) δ 174.0, 170.9, 170.3, 168.9, 168.5, 168.0, 156.2, 134.8, 129.1, 128.8, 128.4, 128.0, 126.8, 80.4, 64.7, 52.4, 51.7, 49.5, 43.1, 41.2, 41.1, 28.2, 27.2, 20.9, 19.6. HRMS (ESI) *m/z*: calcd. for $[M+Na]^+$ $C_{28}H_{42}N_6O_9Na$: 629.2911; found: 629.2905.

Compound 15c

To a solution of ester **11c** (0.803 mmol, 0.323 g) in THF/H_2O (2:1, 39 mL) was added LiOH (0.401 mmol, 0.096 g) at 0 ºC. The reaction was stirred for 2 h at 0 ºC, acidified with a 2 M solution of $NaHSO_4$ to pH 2 and extracted three times with ethyl acetate. The combined organic phases were dried with sodium sulfate

and concentrated to yield the respective acid **13c** (0.775 mmol, 0.301 g, 96% yield), which was used without further purification. To a solution of benzylamine (1.55 mmol, 0.166 g) in methanol (10 mL) were added sodium sulfate (0.20 g) and paraformaldehyde (1.55 mmol, 0.0465 g) and stirring was continued for 1 h at rt. Acid **13c** (0.775 mmol, 0.301 g) was added and after 10 min methyl isocyanoacetate (0.775 mmol, 0.070 mL) was added. The reaction was stirred for 24 h at rt. After filtration, the solution was concentrated in vacuum and the residue was purified by column chromatography ($CH_2Cl_2 \rightarrow$ 4% MeOH/CH_2Cl_2) to yield compound **15c** (0.425 mmol, 0.258 g, 55% yield) as a yellow solid. R_f (CH_2Cl_2/MeOH 10%) = 0.44. m.p = 84-86 ºC. ^{1}H NMR (600 MHz, $CDCl_3$, presence of rotamers) δ 9.83 (br s, 1H), 8.58 (br s, 1H), 7.48 and 7.34 (2 br s, 1H), 7.28 and 7.10 (m, 6H), 5.87 and 5.78 (br s, 1H), 4.71-4.54 (m, 2H), 4.17-3.82 (m, 8H), 3.63 and 3.61 (2s, 3H), 2.33-2.26 and 2.16-2.09 (2m, 2H), 1.37-1.20 (m, 15H), 0.89-0.86 (m, 3H). ^{13}C NMR (150 MHz, $CDCl_3$, presence of rotamers) δ 175.2, 172.0, 170.4, 170.1, 169.2, 168.6, 156.5, 135.3, 129.1, 128.7, 128.4, 128.0, 126.8, 80.6, 64.6, 52.3, 50.0, 49.4, 42.9, 41.8, 41.1, 28.3, 26.5, 24.4, 22.6, 8.4. HRMS (ESI) *m/z*: calcd. for $[M+Na]^+$ $C_{28}H_{42}N_6O_9Na$: 629.2911; found: 629.2903.

References

1. Khan, K. M.; Rasheed, M.; Ullah, Z.; Hayat, S.; Kaukab, F.; Choudhary, M. I.; Rahman, A.-U.; Perveen, S. *Bioorg. Med. Chem*. **2003**, *11*, 1381-1387.
2. Caplar, V.; Zinir, M.; Pozzo, J.-L.; Fages, F.; Mieden-Gundert, G.; Vogtle, F. *Eur. J. Org. Chem*. **2004**, 4048-4059.
3. Ottersbach, P. A.; Schnakenburg, G.; Gütschow, M. *Chem. Commun*. **2012**, *48*, 5772-5774.
4. Gaccioli, F.; Franchi-Gazzola, R.; Lanfranchi, M.; Marchio, L.; Metta, G.; Pellinghelli, M. A.; Tardito, S.; Tegoni, M. *J. Inorg. Biochem*. **2005**, *99*, 1573-1584.

Spectra of compounds

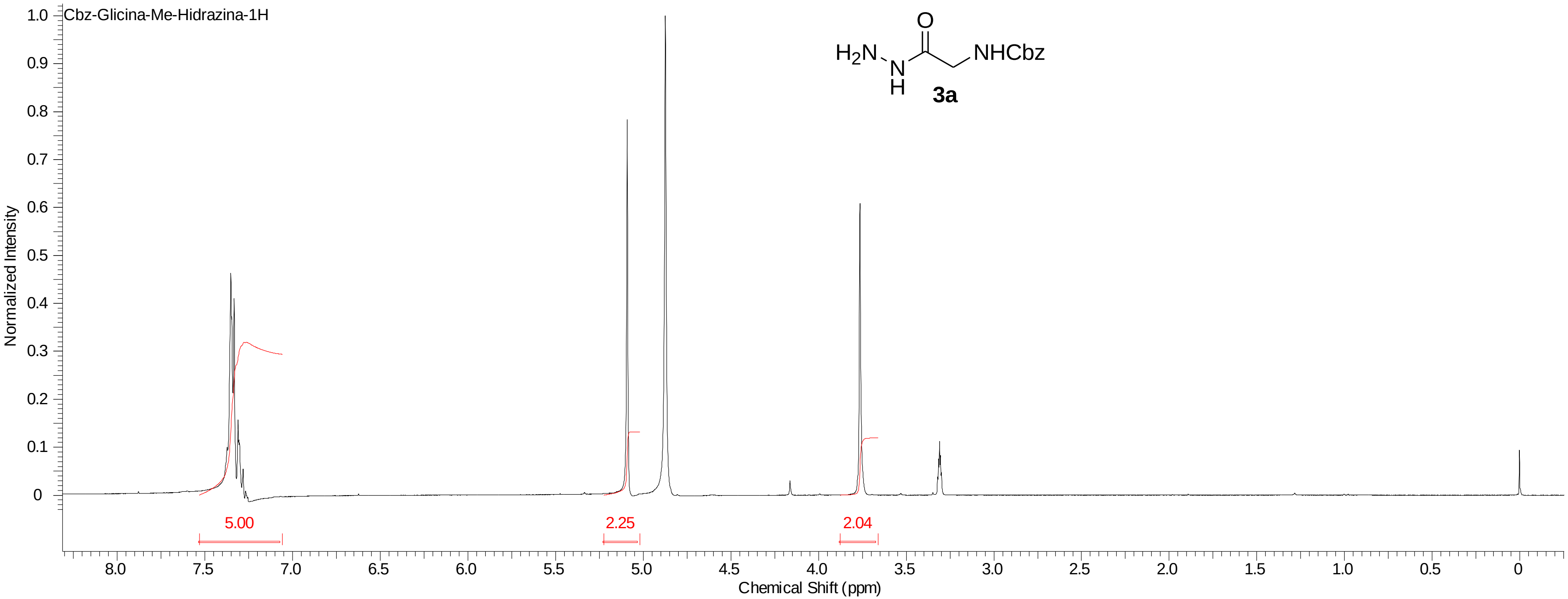

Figure S1: ^{1}H NMR (300 MHz, CD_3OD) spectrum of compound **3a**.

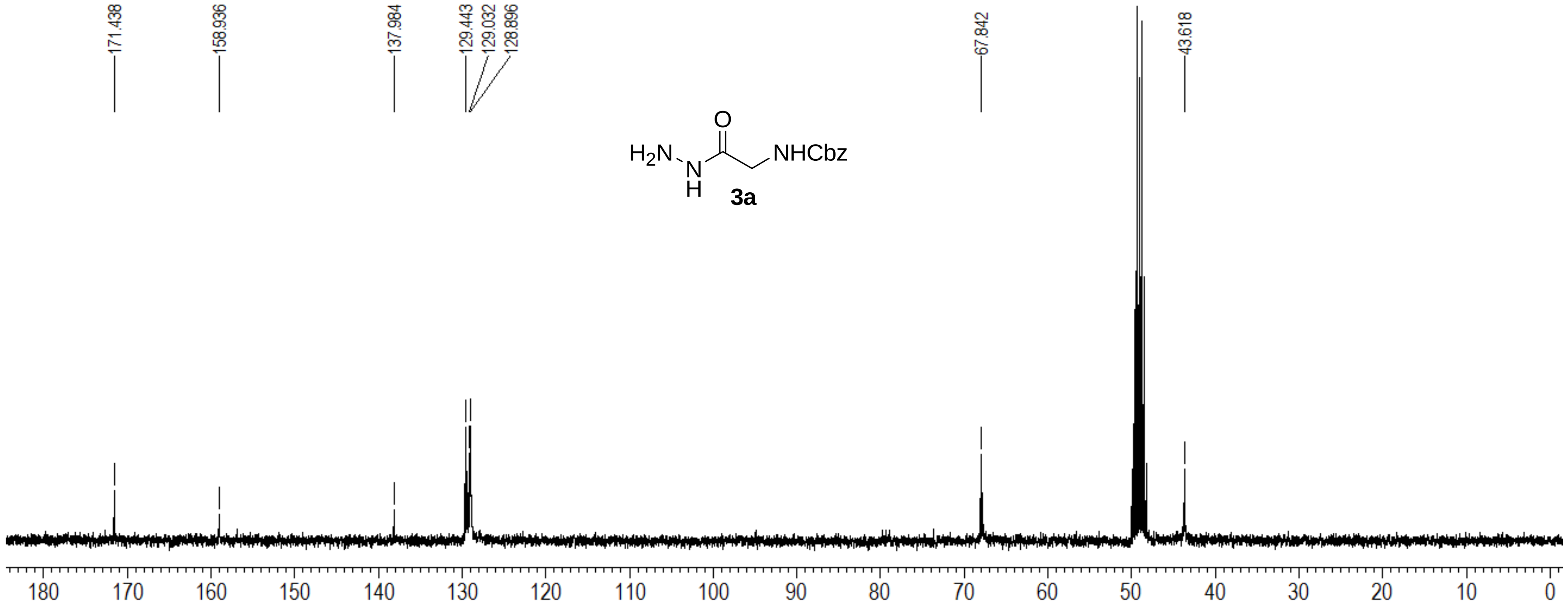

Figure S2: ^{13}C NMR (75.46 MHz, CD_3OD) spectrum of compound **3a**.

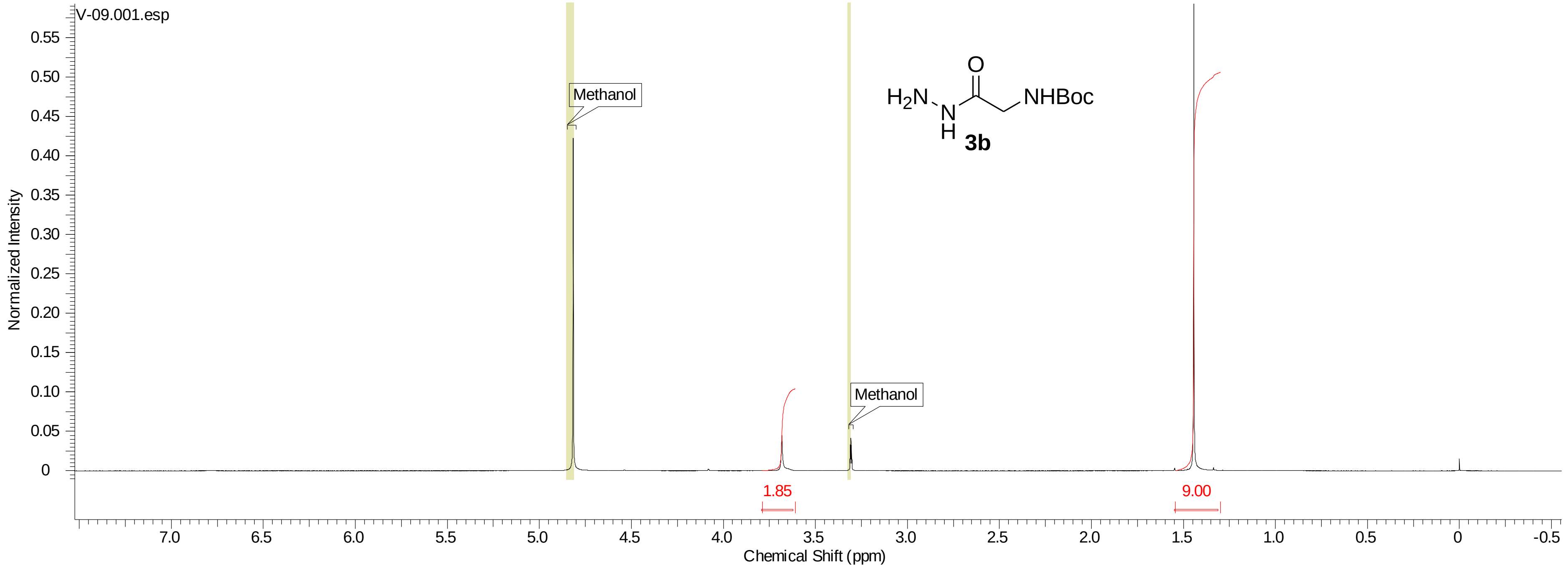

Figure S3: ^{1}H NMR (300 MHz, CD_3OD) spectrum of compound **3b**.

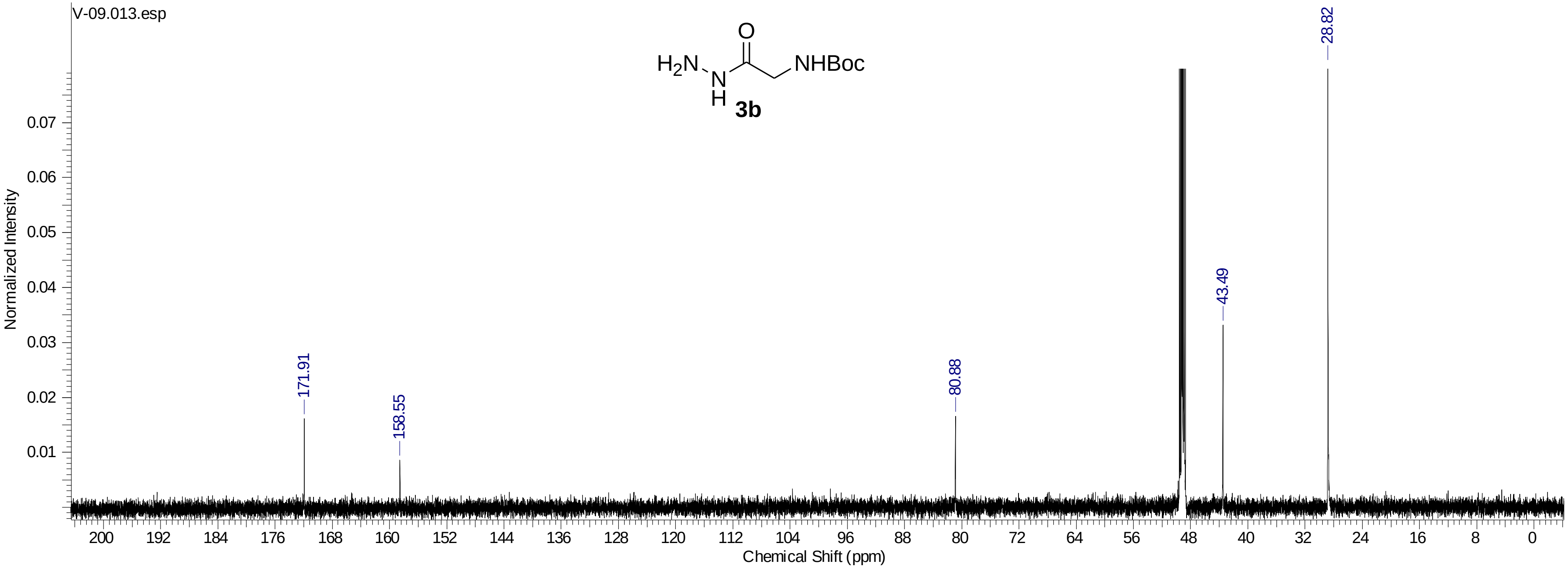

Figure S4: ^{13}C NMR (75.46 MHz, CD_3OD) spectrum of compound **3b**.

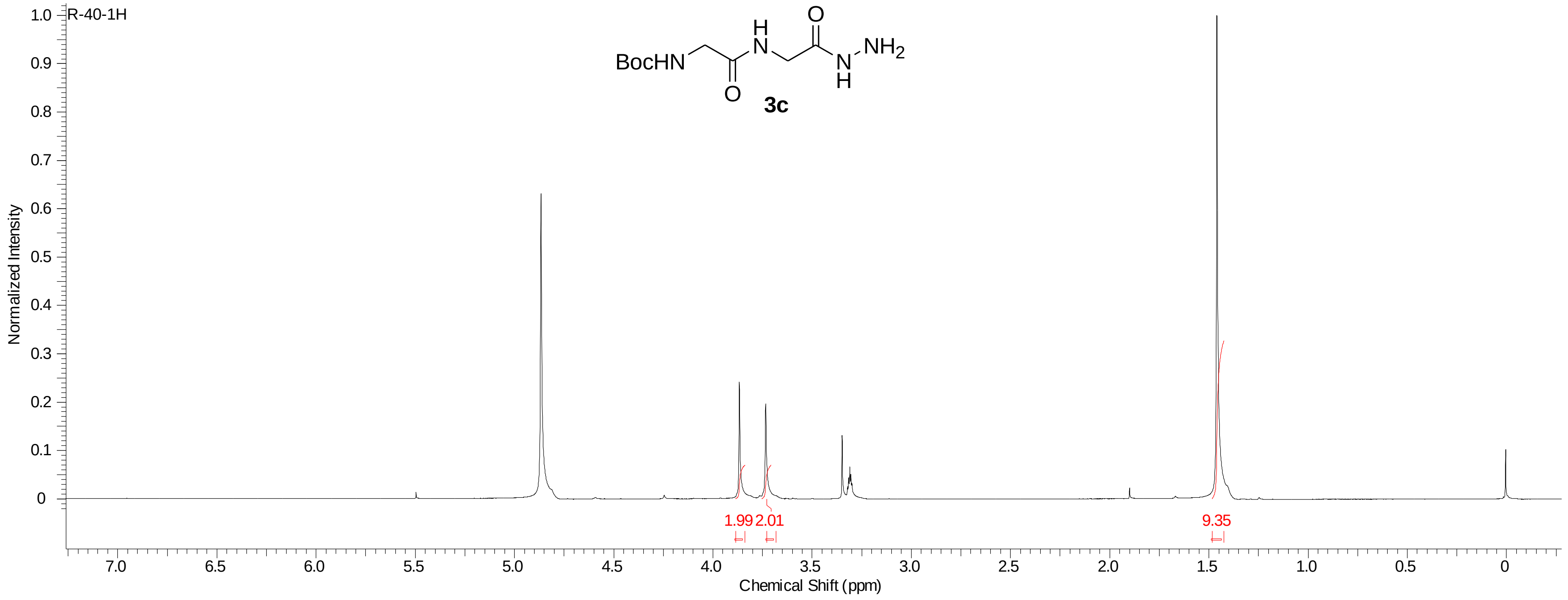

Figure S5: ^{1}H NMR (300 MHz, CD_3OD) spectrum of compound **3c**.

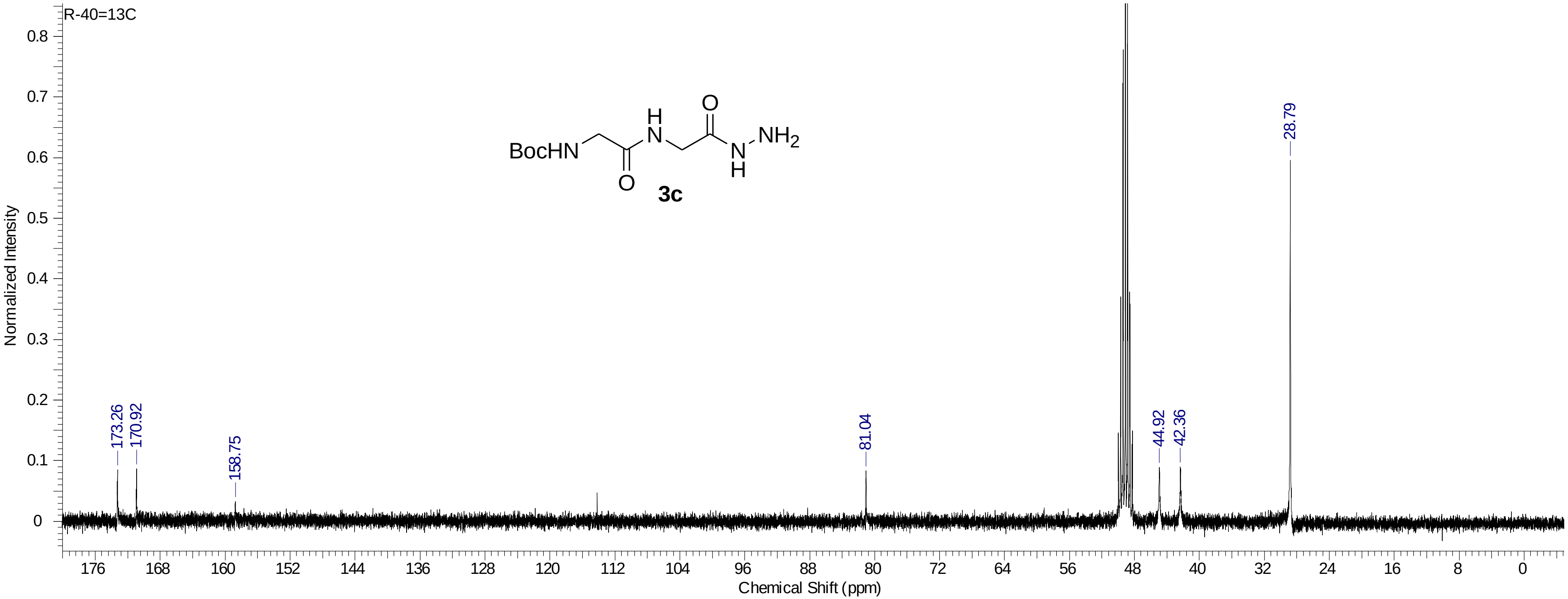

Figure S6: ^{13}C NMR (75.46 MHz, CD_3OD) spectrum of compound **3c**.

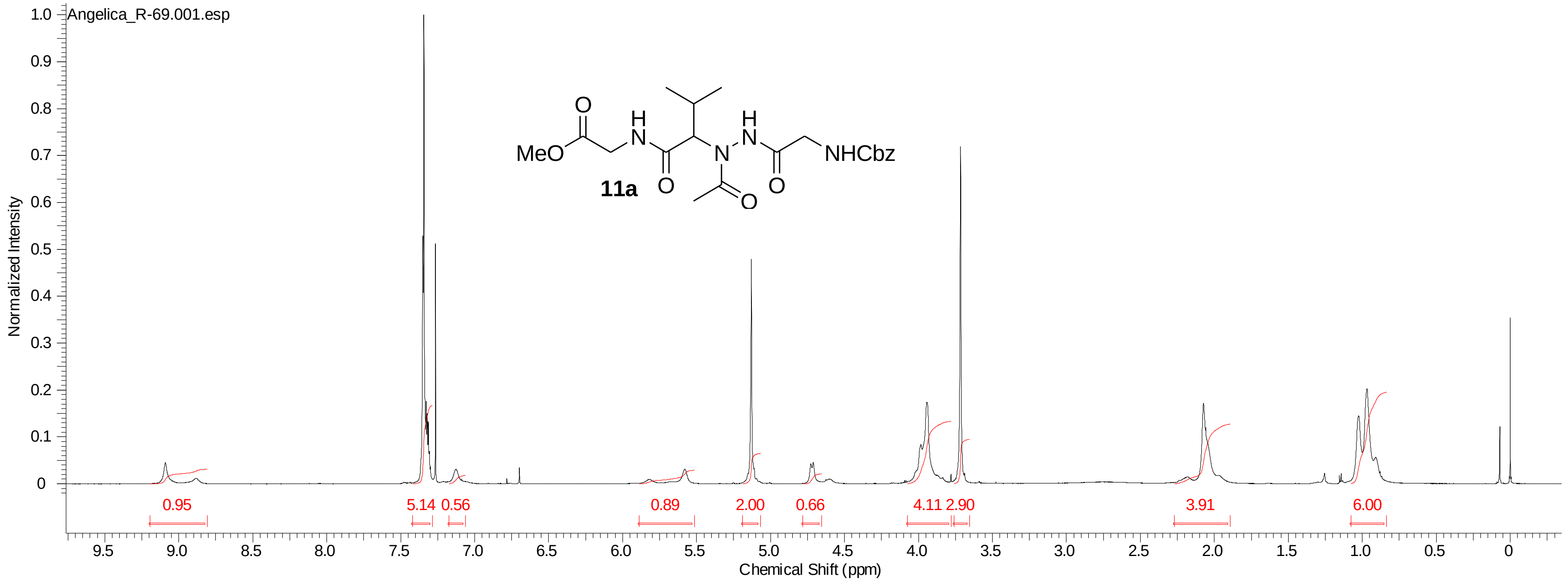

Figure S7: ^{1}H NMR (300 MHz, $CDCl_3$, presence of rotamers) spectrum of compound **11a**.

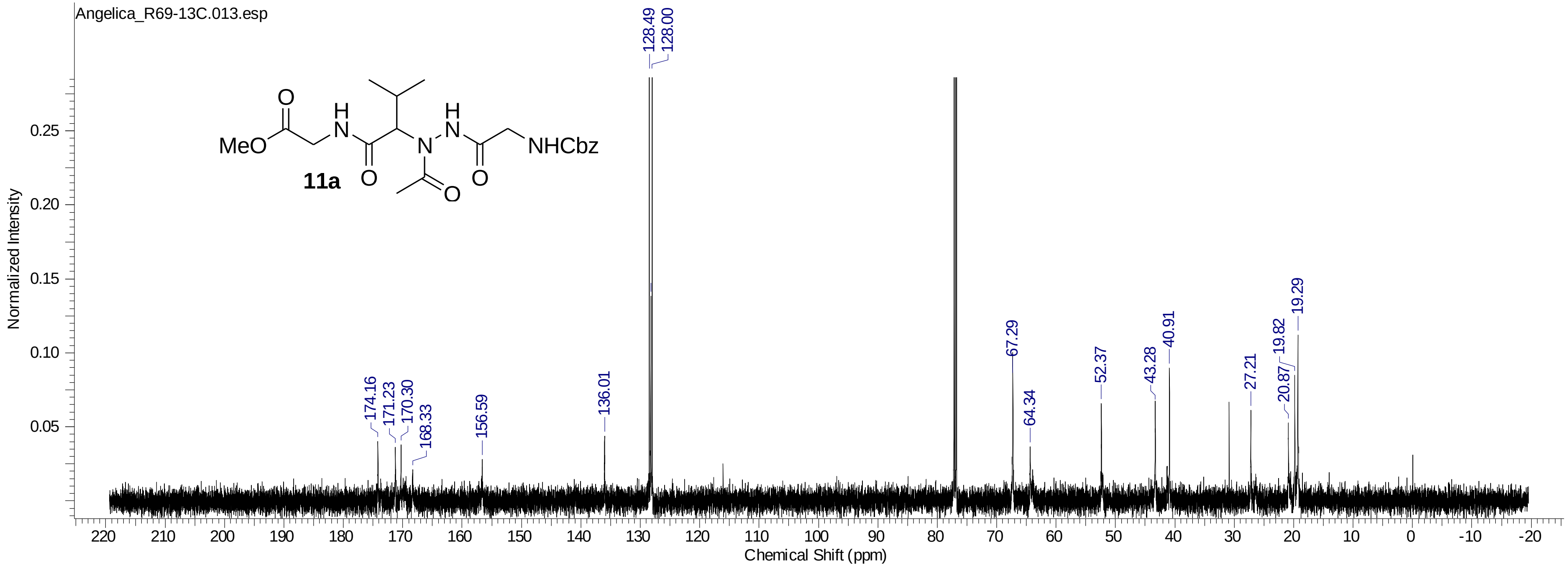

Figure S8: ^{13}C NMR (75.46 MHz, $CDCl_3$, presence of rotamers) spectrum of compound **11a**.

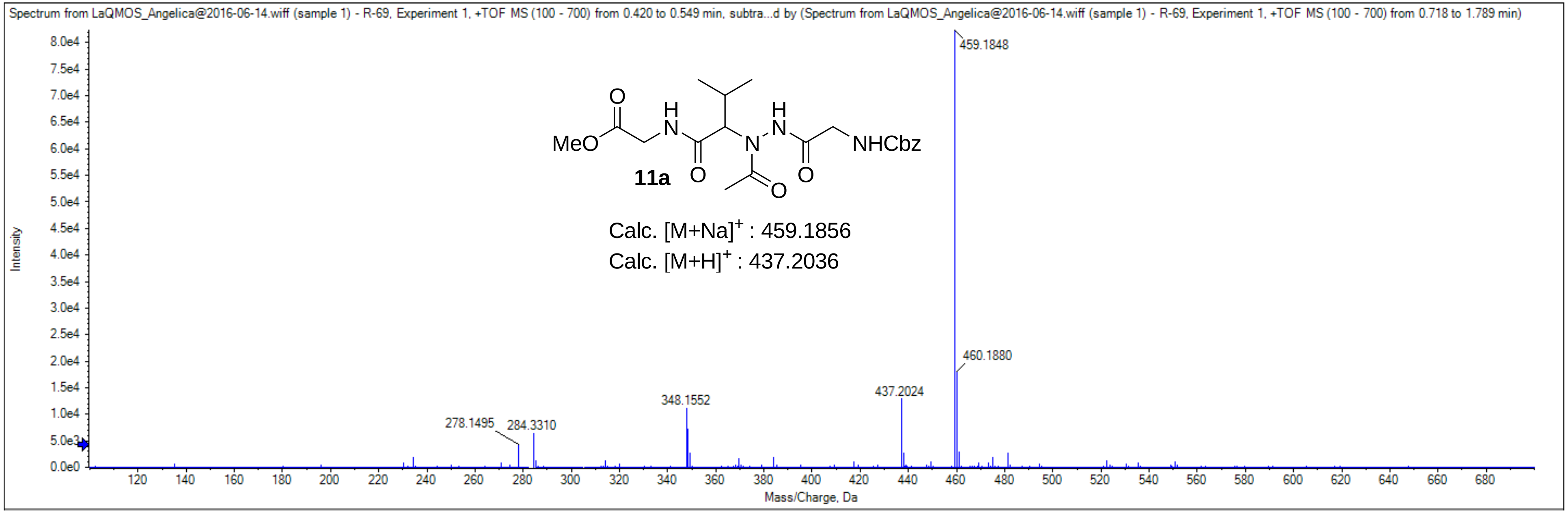

Figure S9: ESI-HRMS of compound **11a.**

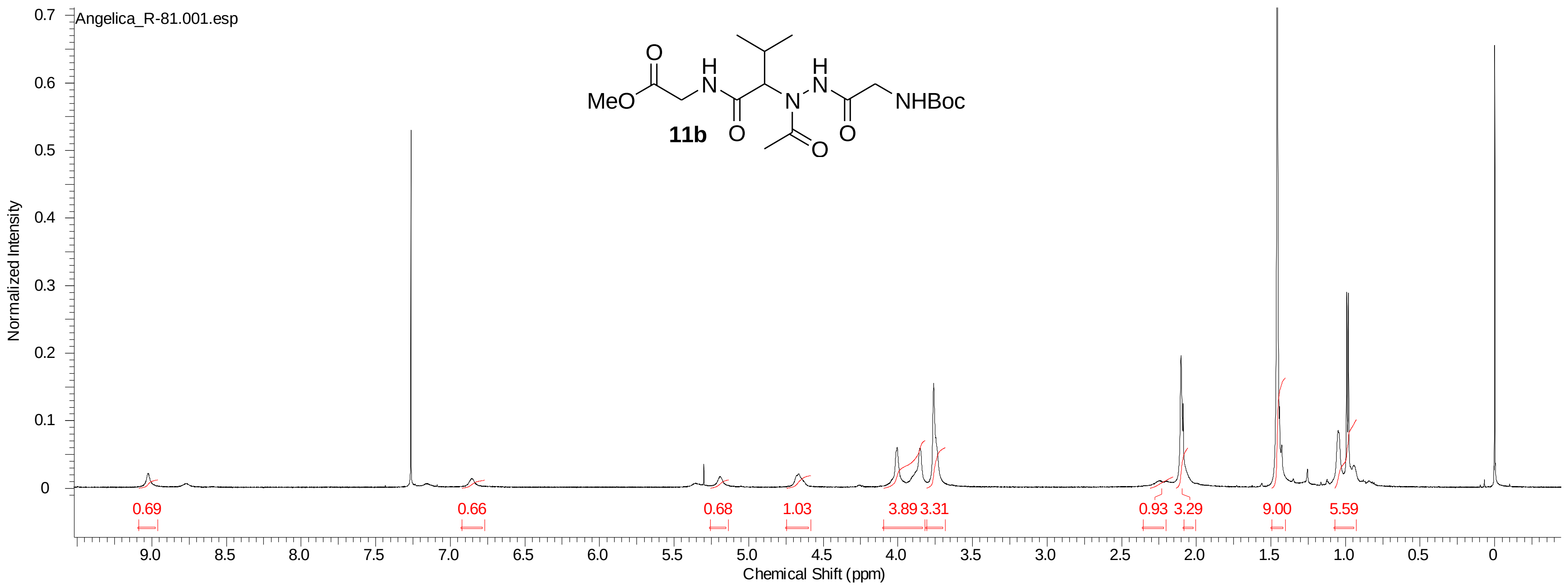

Figure S10: ^{1}H NMR (300 MHz, $CDCl_3$, presence of rotamers) spectrum of compound **11b**.

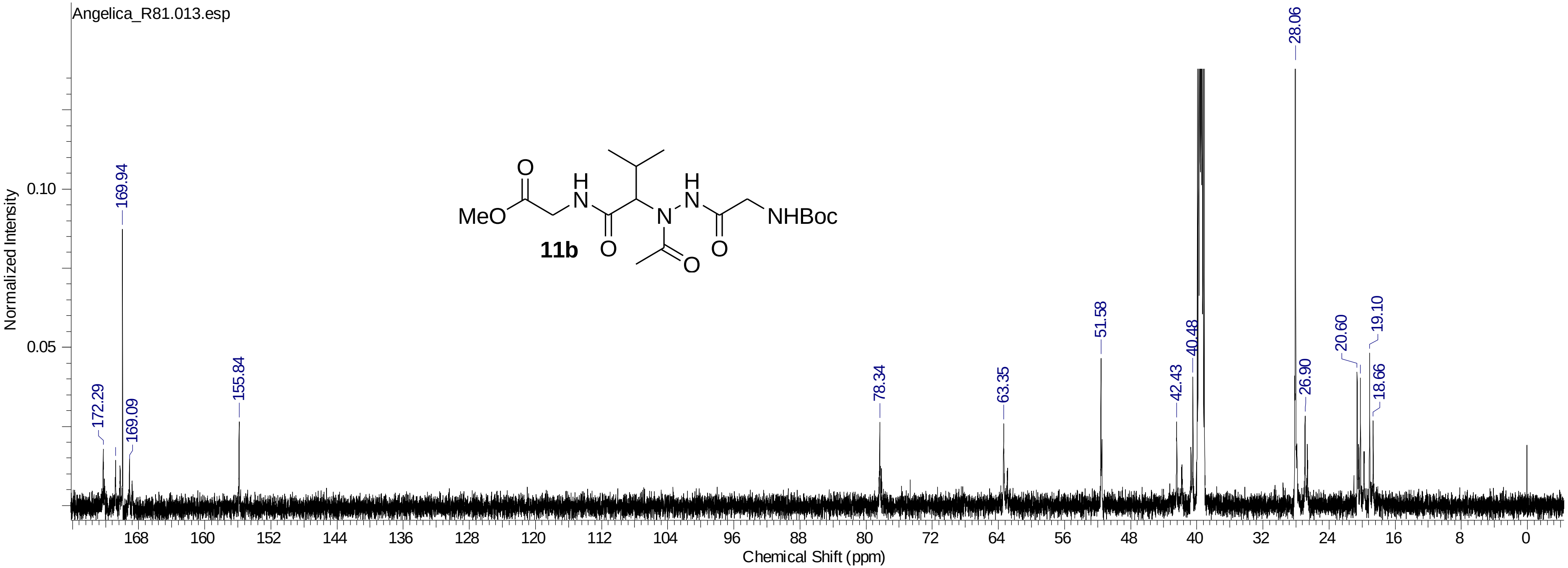

Figure S11: ^{13}C NMR (75.46 MHz, DMSO-d_6, presence of rotamers) spectrum of compound **11b**.

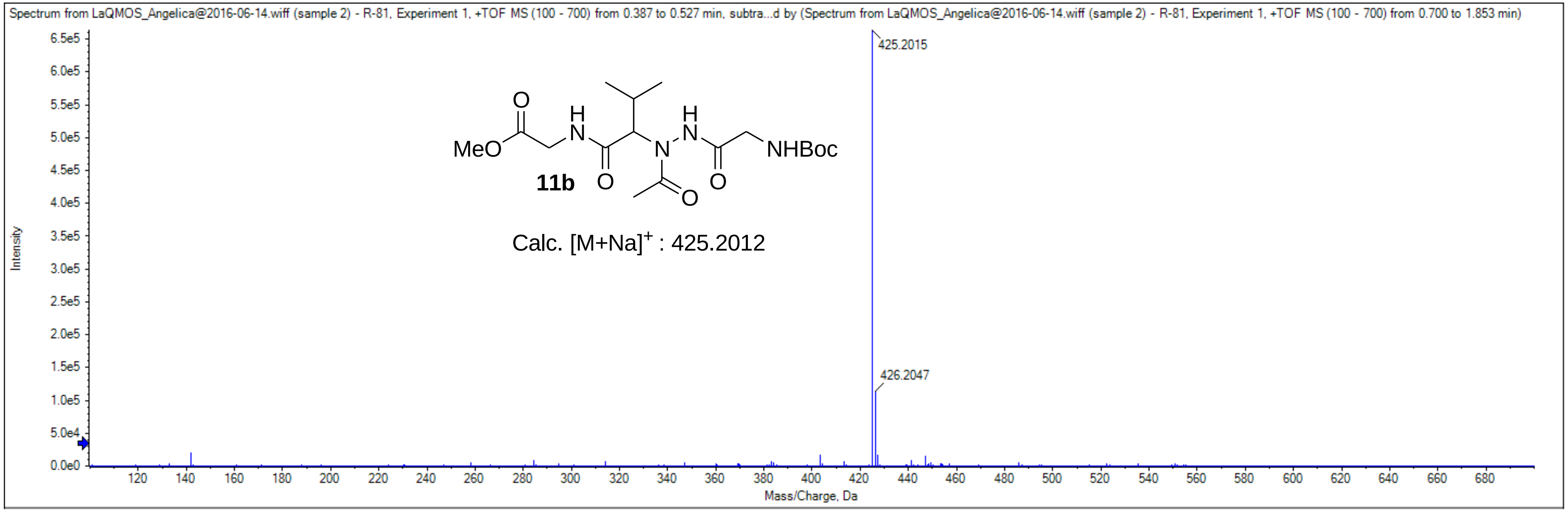

Figure S12: ESI-HRMS of compound **11b.**

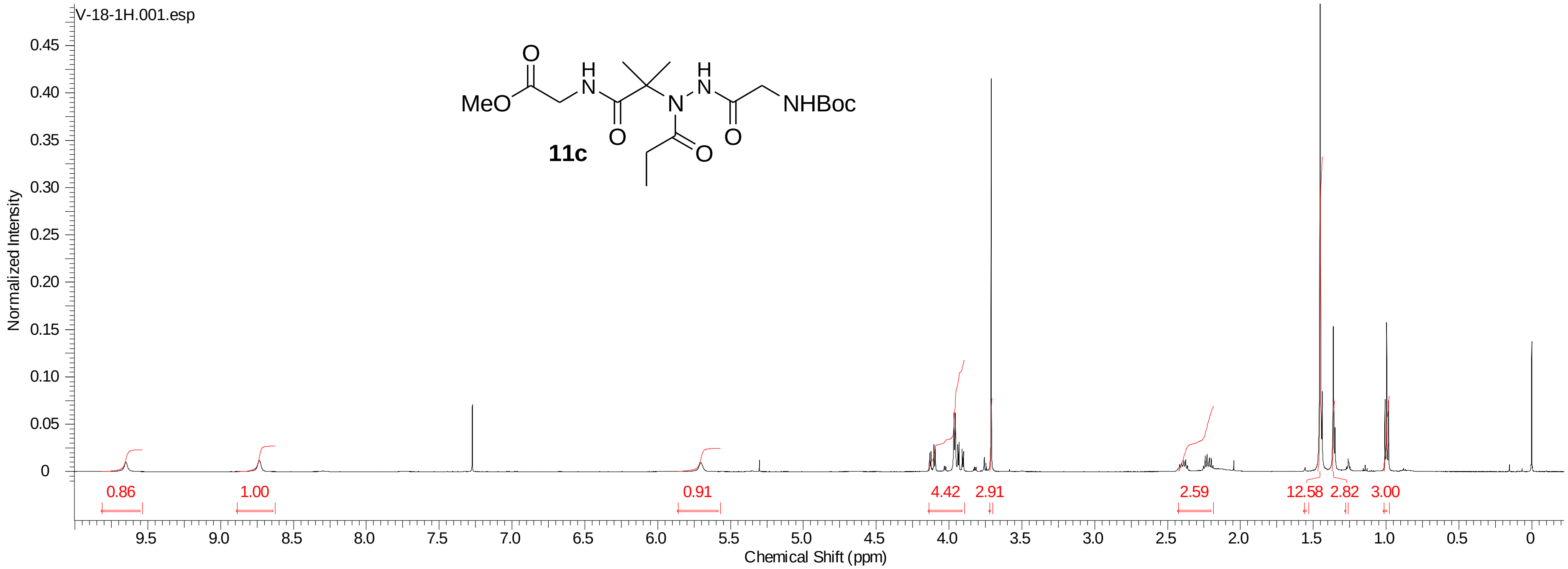

Figure S13: ^{1}H NMR (600 MHz, $CDCl_3$, presence of rotamers) spectrum of compound **11c**.

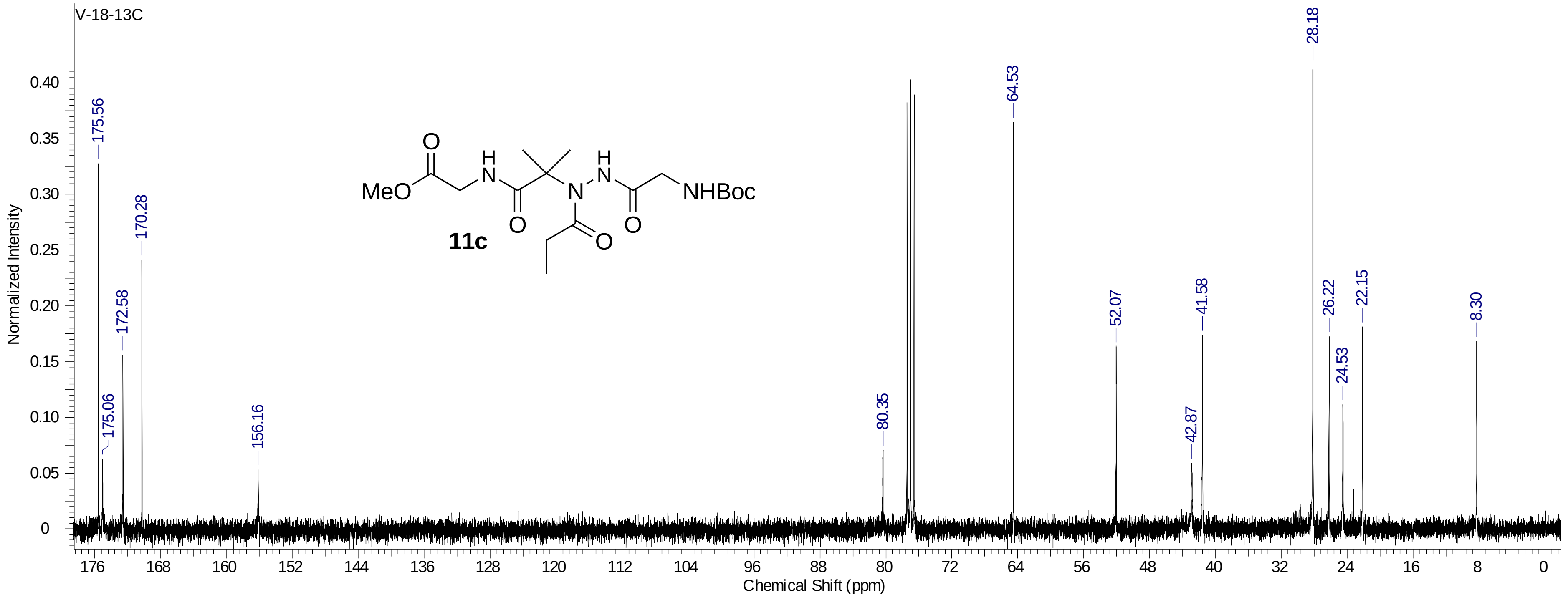

Figure S14: ^{13}C NMR (150 MHz, $CDCl_3$, presence of rotamers) spectrum of compound **11c**.

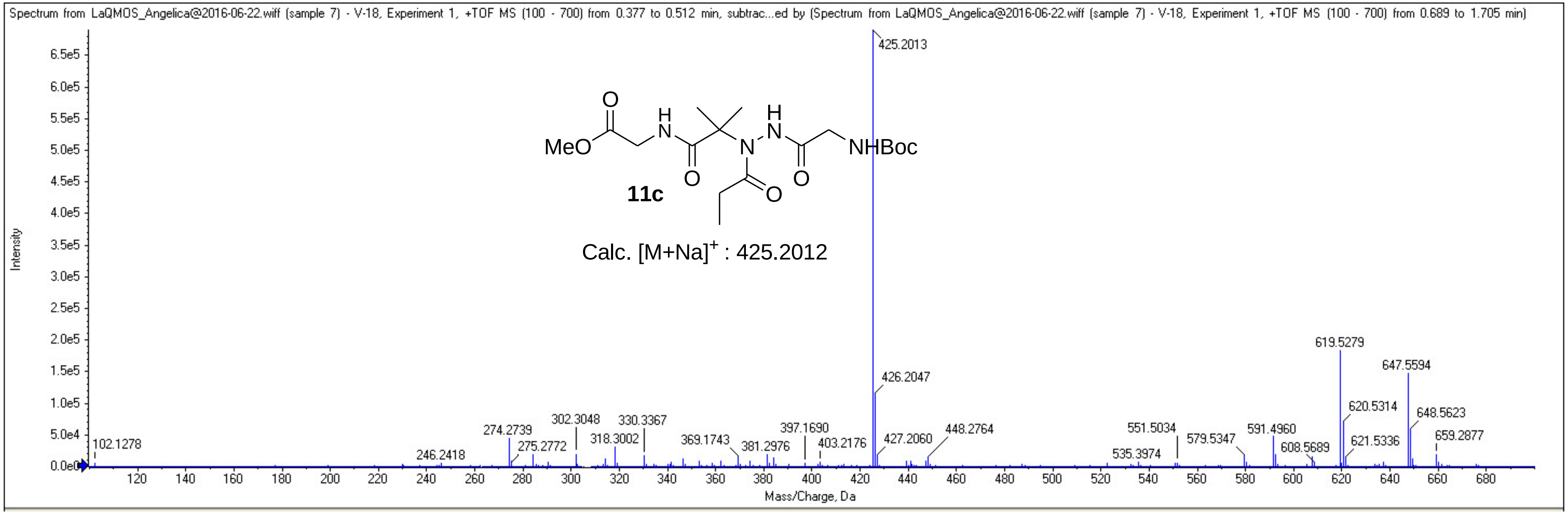

Figure S15: ESI-HRMS of compound **11c.**

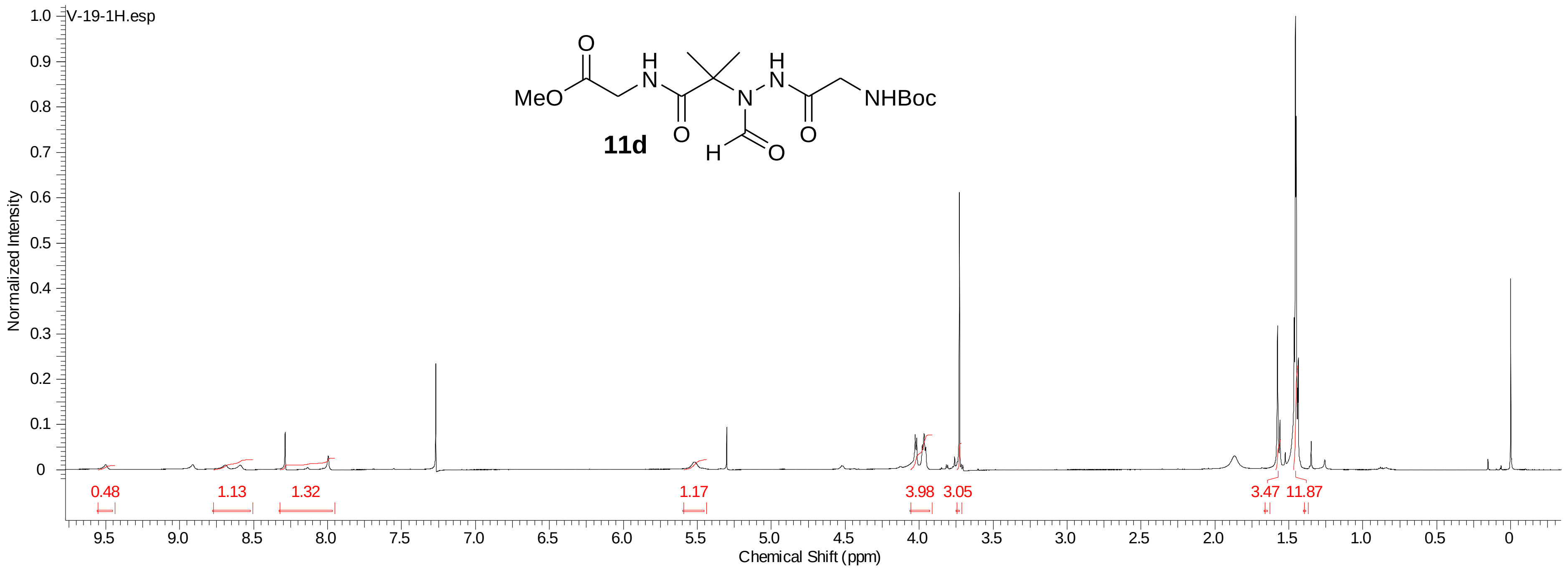

Figure S16: ^{1}H NMR (600 MHz, $CDCl_3$, presence of rotamers) spectrum of compound **11d**.

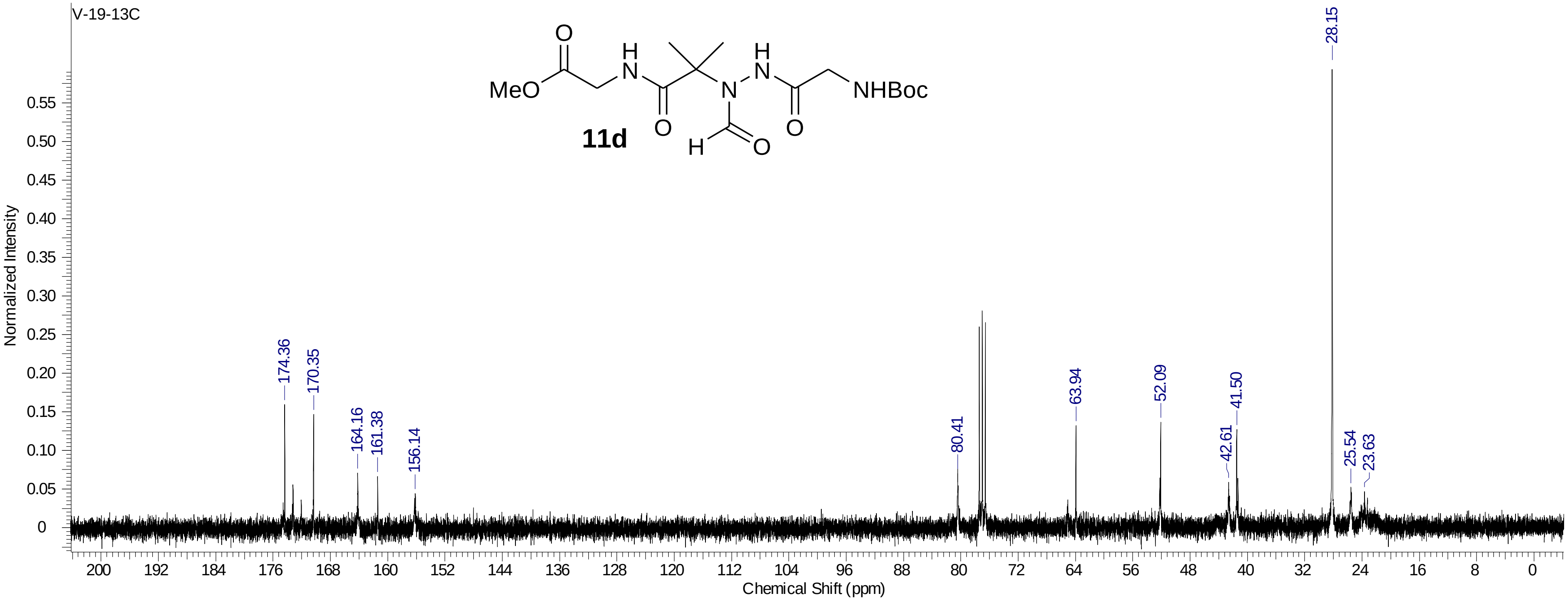

Figure S17: ^{13}C NMR (150 MHz, $CDCl_3$, presence of rotamers) spectrum of compound **11d**.

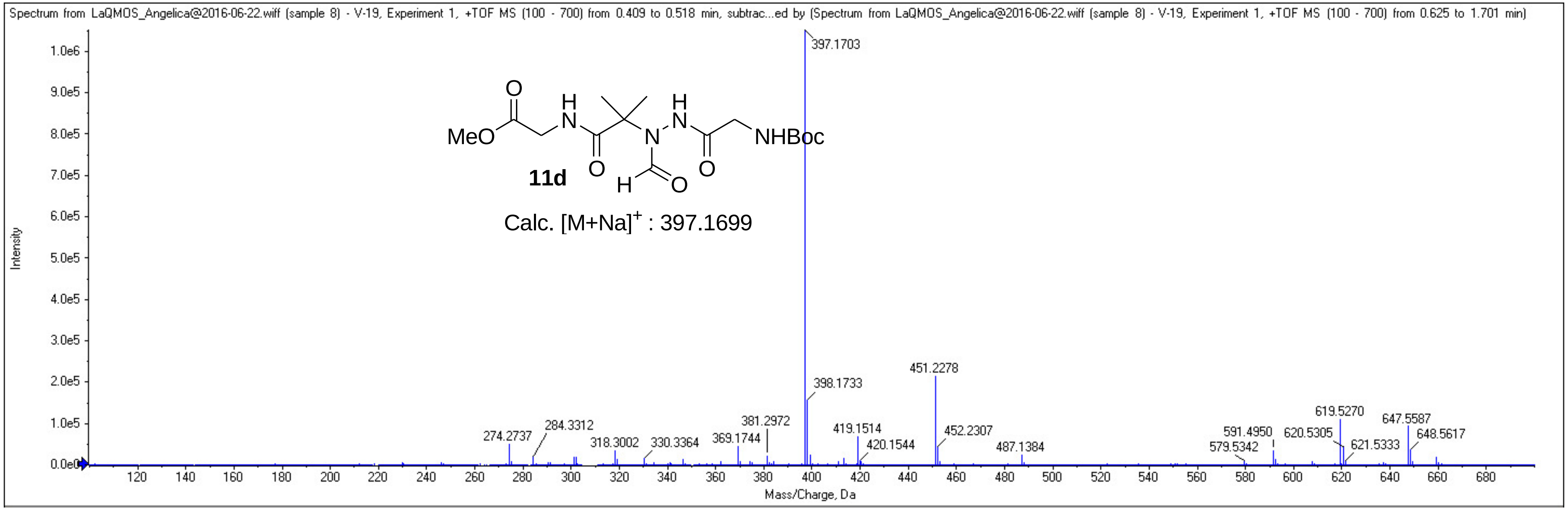

Figure S18: ESI-HRMS of compound **11d.**

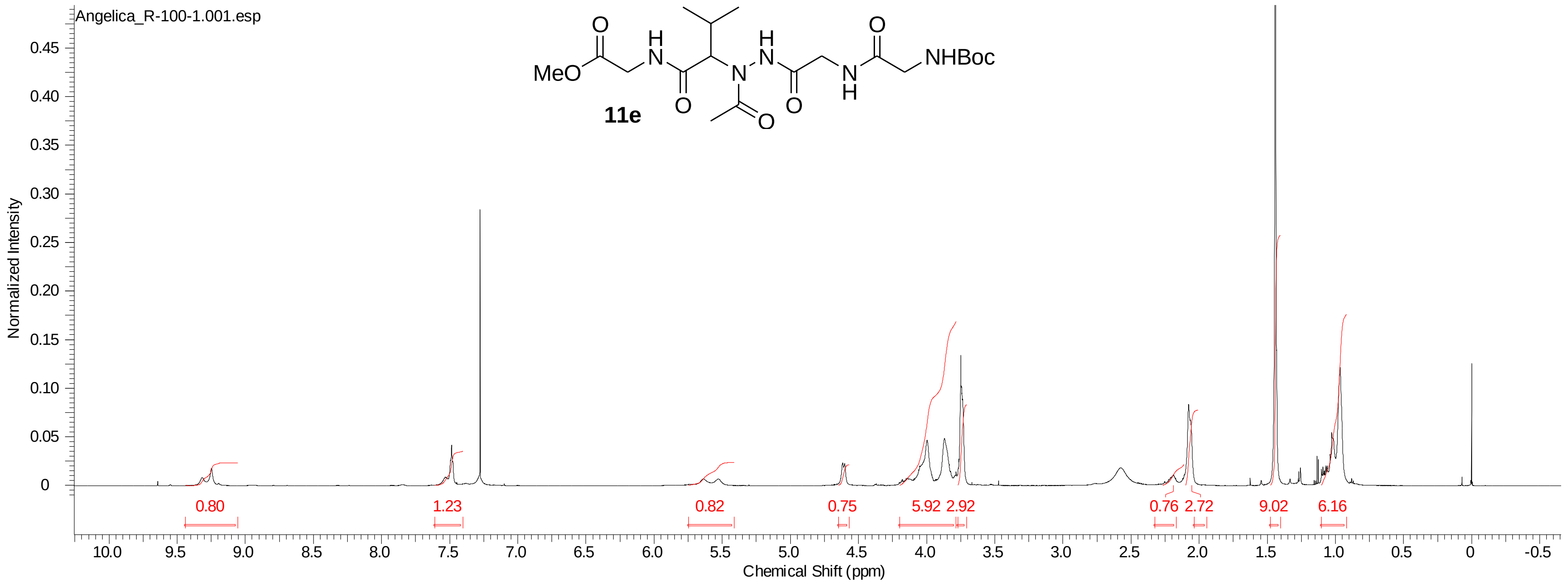

Figure S19: ^{1}H NMR (600 MHz, $CDCl_3$, presence of rotamers) spectrum of compound **11e**.

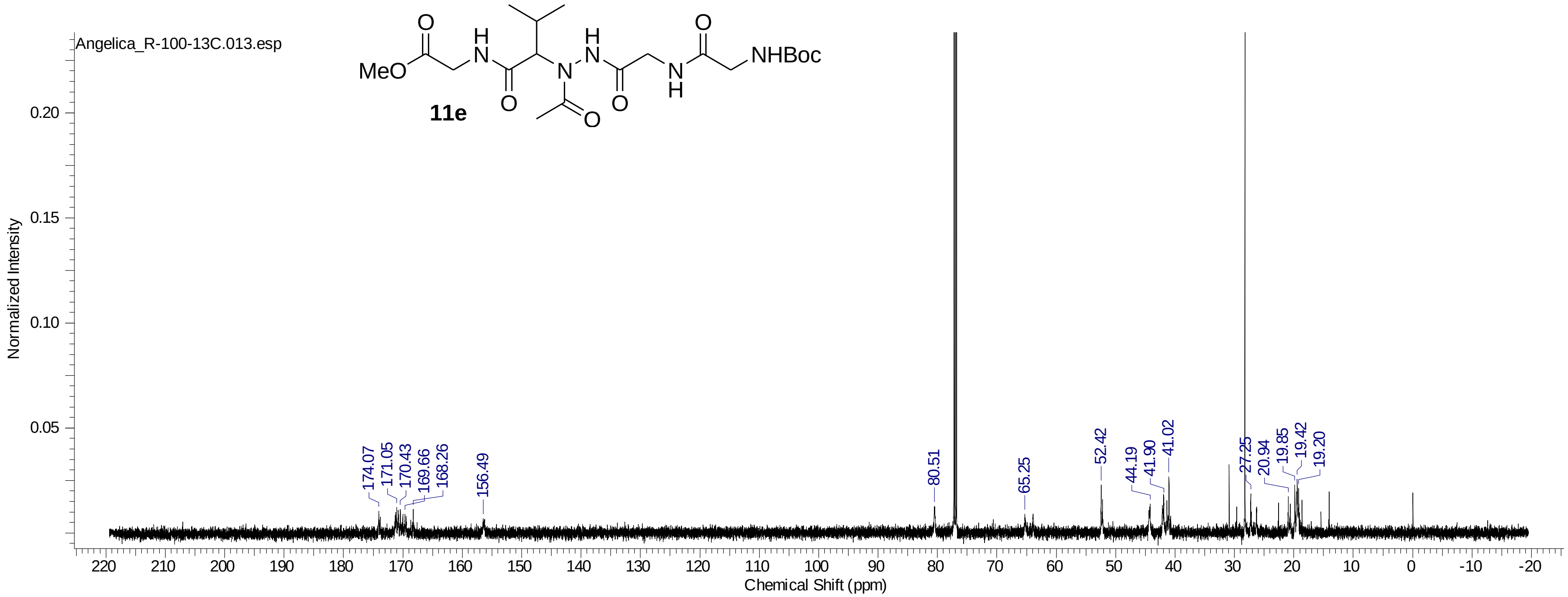

Figure S20: ^{13}C NMR (150 MHz, $CDCl_3$, presence of rotamers) spectrum of compound **11e**.

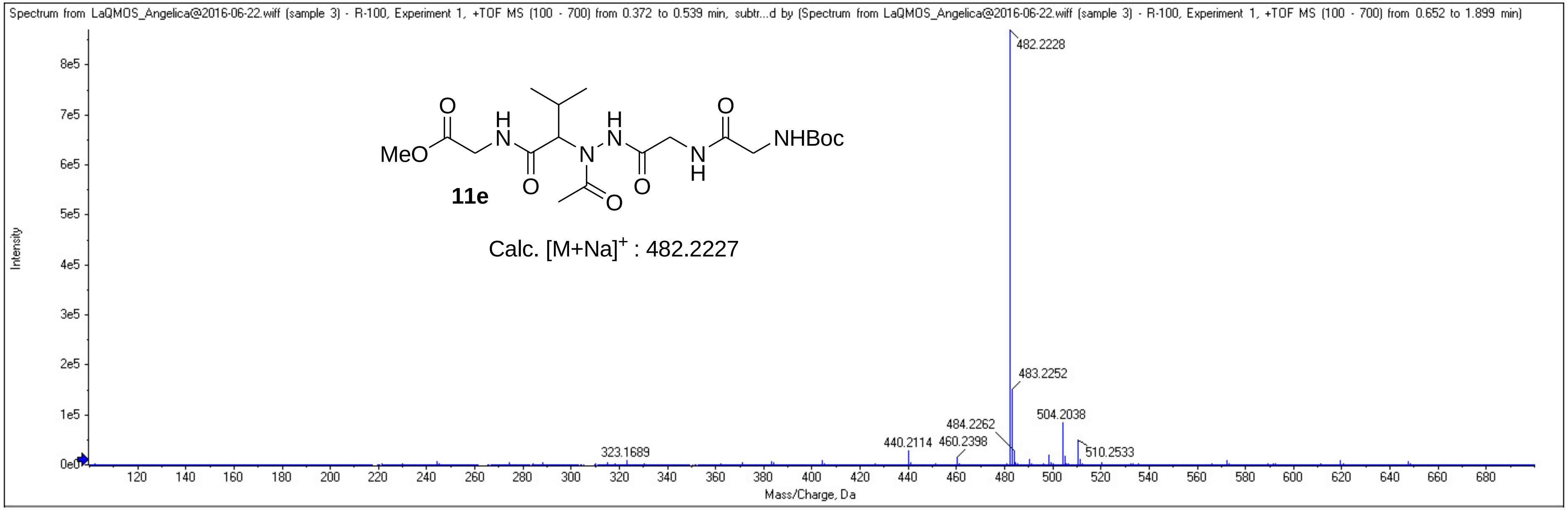

Figure S21: ESI-HRMS of compound **11e.**

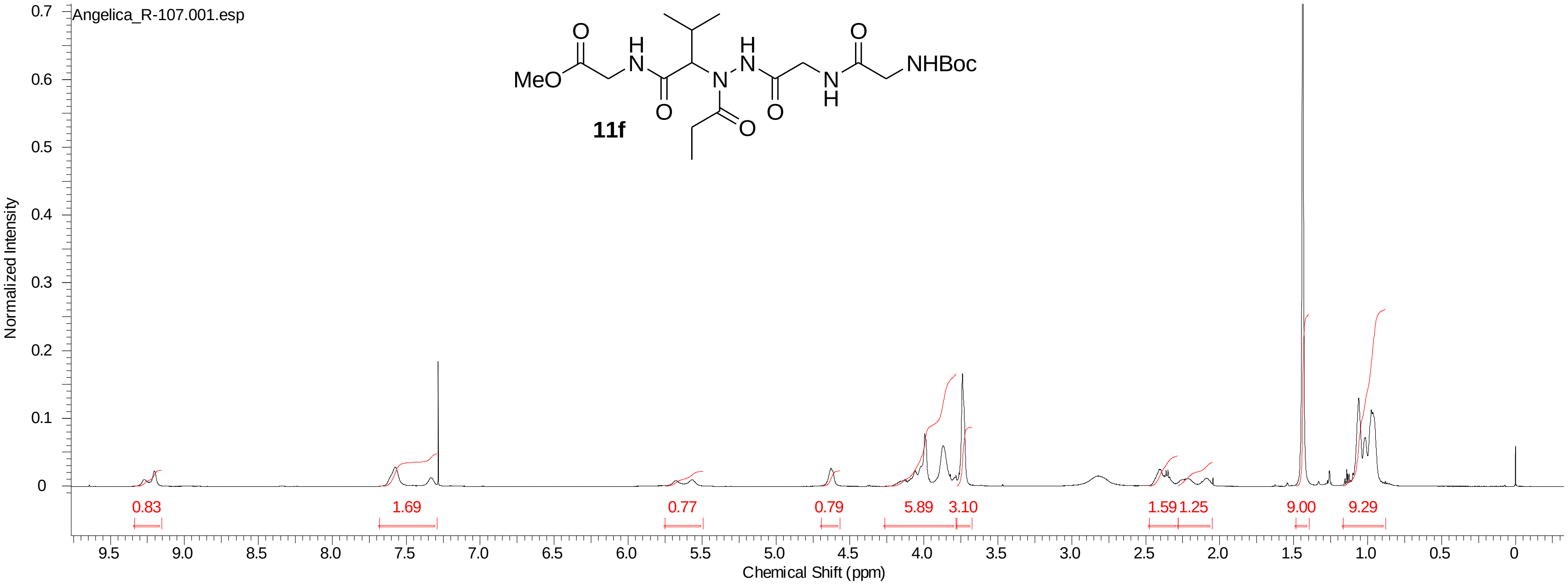

Figure S22: ^{1}H NMR (600 MHz, $CDCl_3$, presence of rotamers) spectrum of compound **11f**.

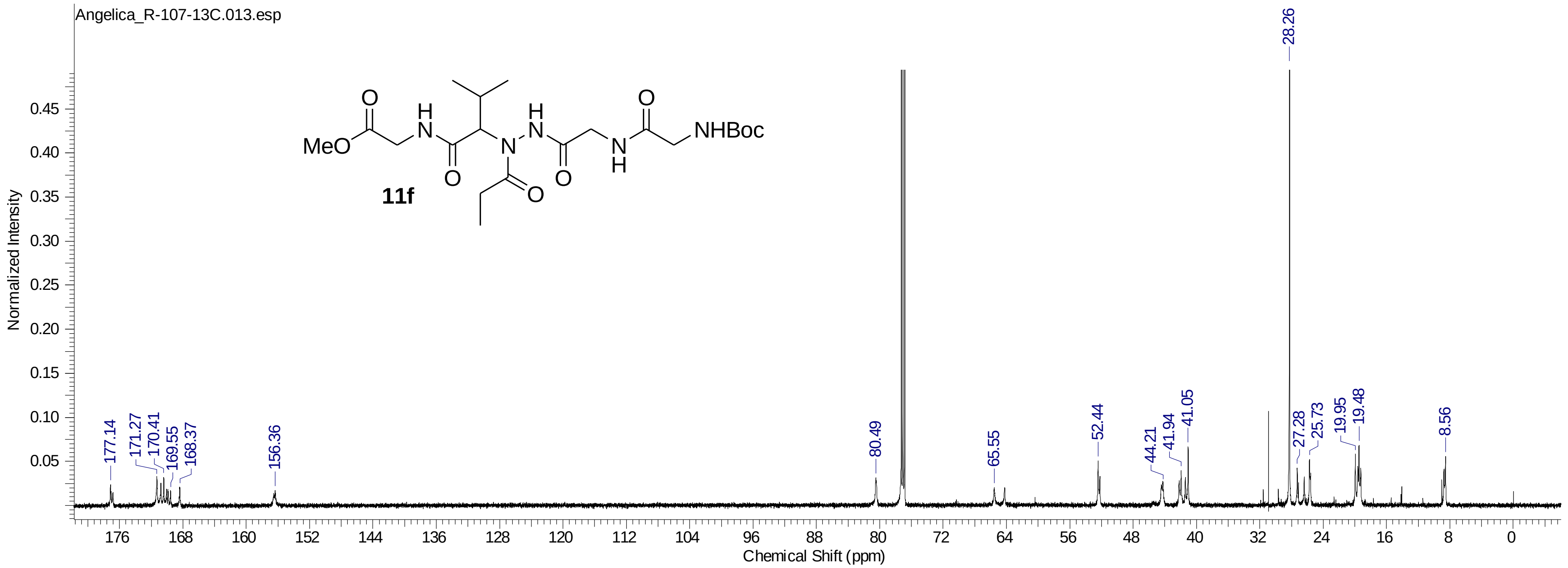

Figure S23: ^{13}C NMR (150 MHz, $CDCl_3$, presence of rotamers) spectrum of compound **11f**.

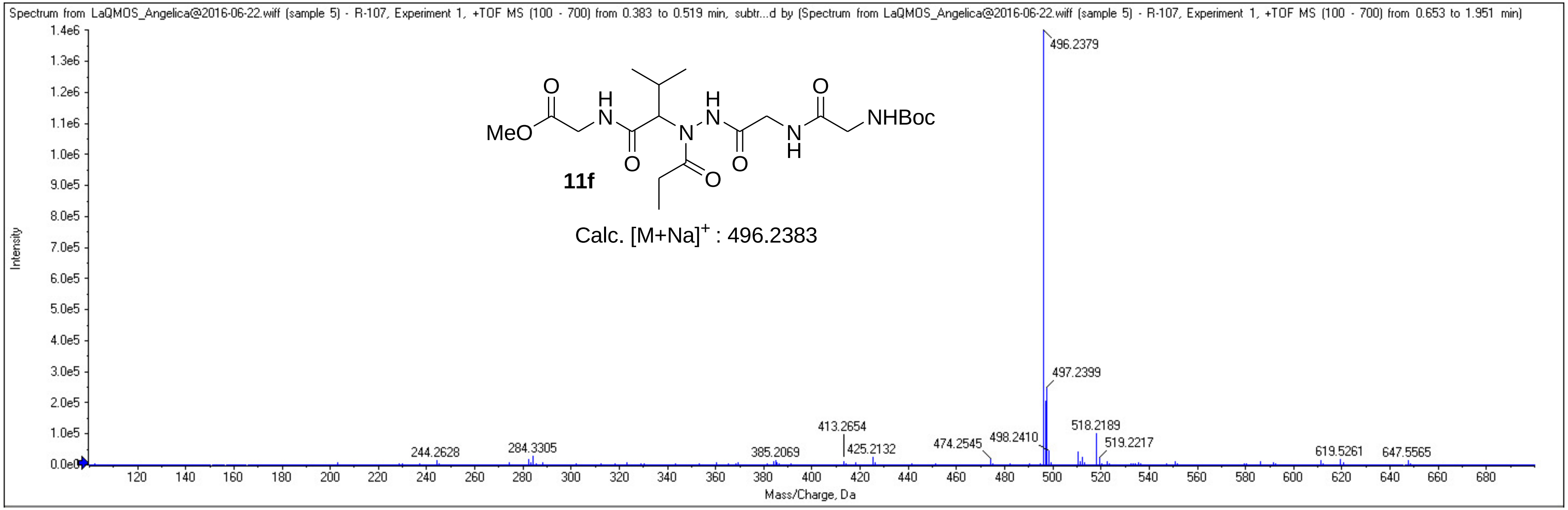

Figure S24: ESI-HRMS of compound **11f.**

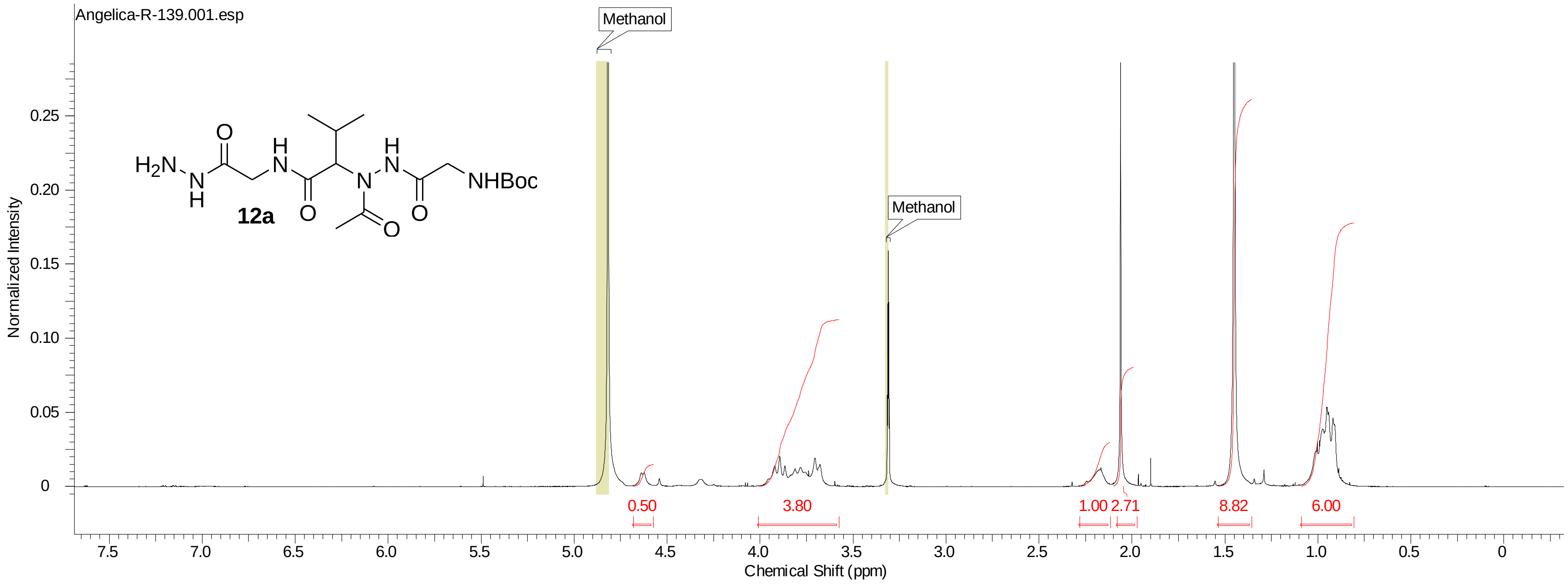

Figure S25: ^{1}H NMR (600 MHz, CD_3OD) spectrum of compound **12a**.

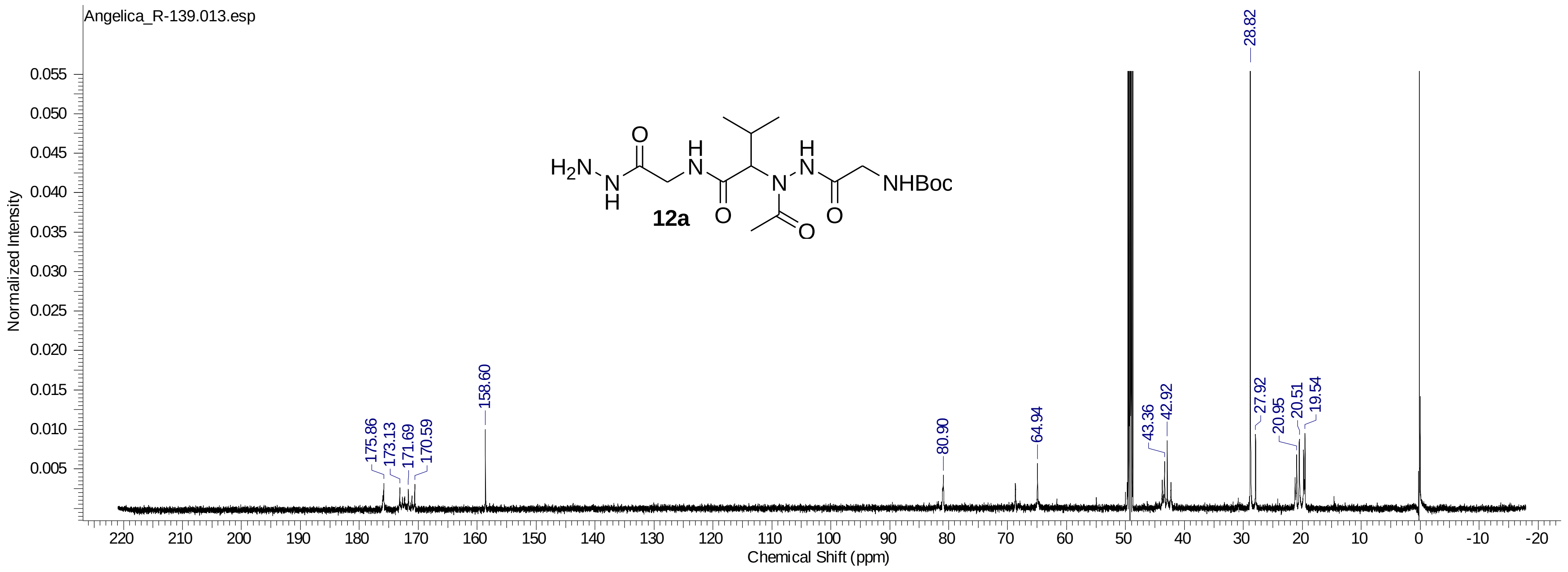

Figure S26: ^{13}C NMR (150 MHz, CD_3OD, presence of rotamers) spectrum of compound **12a**.

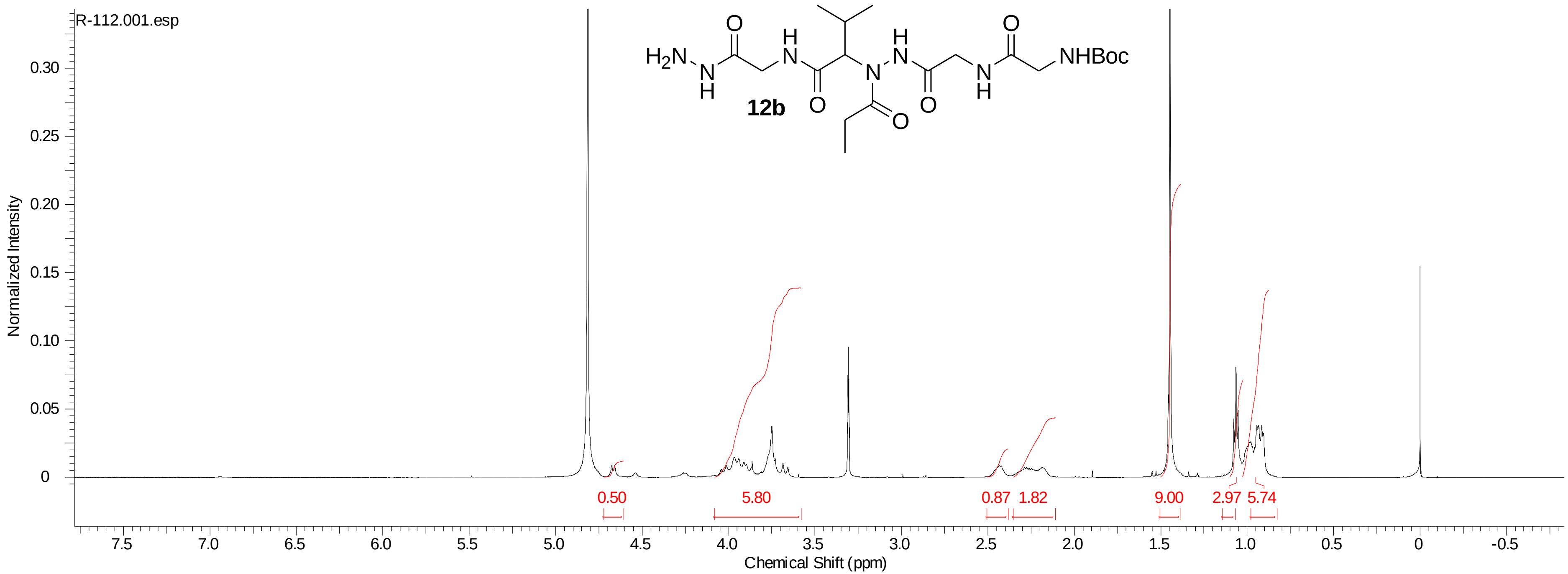

Figure S27: ^{1}H NMR (600 MHz, CD_3OD) spectrum of compound **12b**.

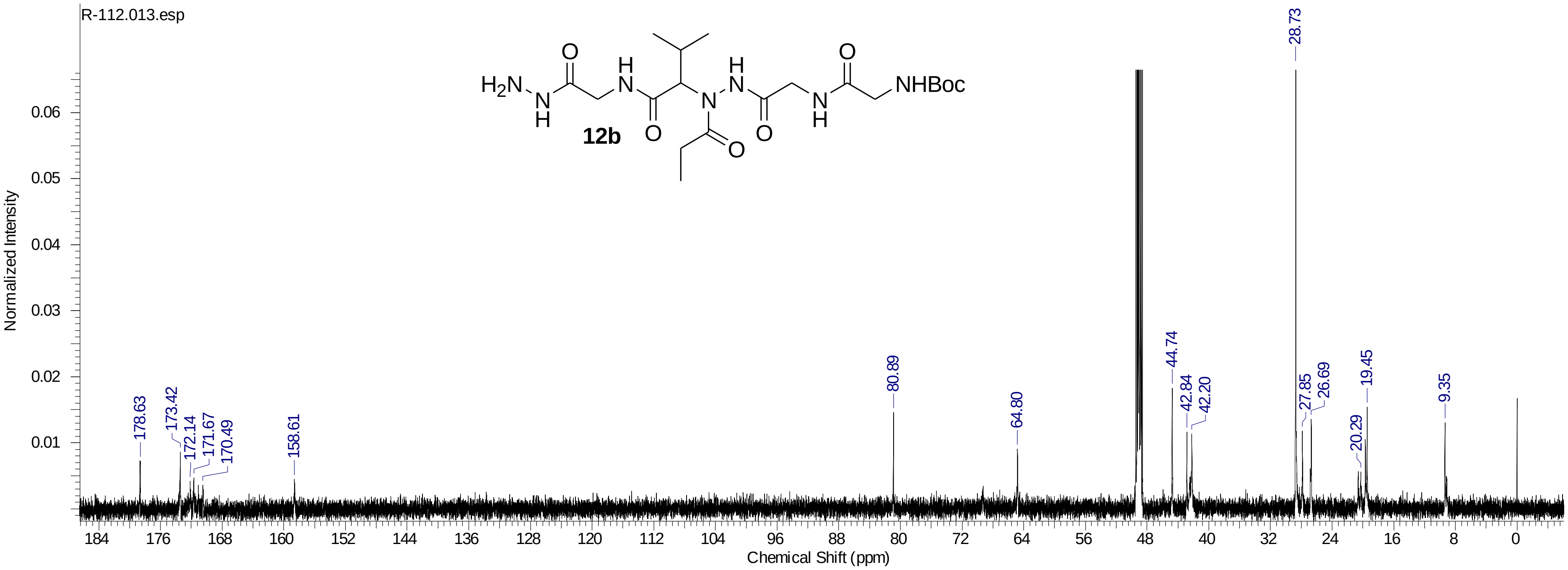

Figure S28: ^{13}C NMR (150 MHz, CD_3OD) spectrum of compound **12b**.

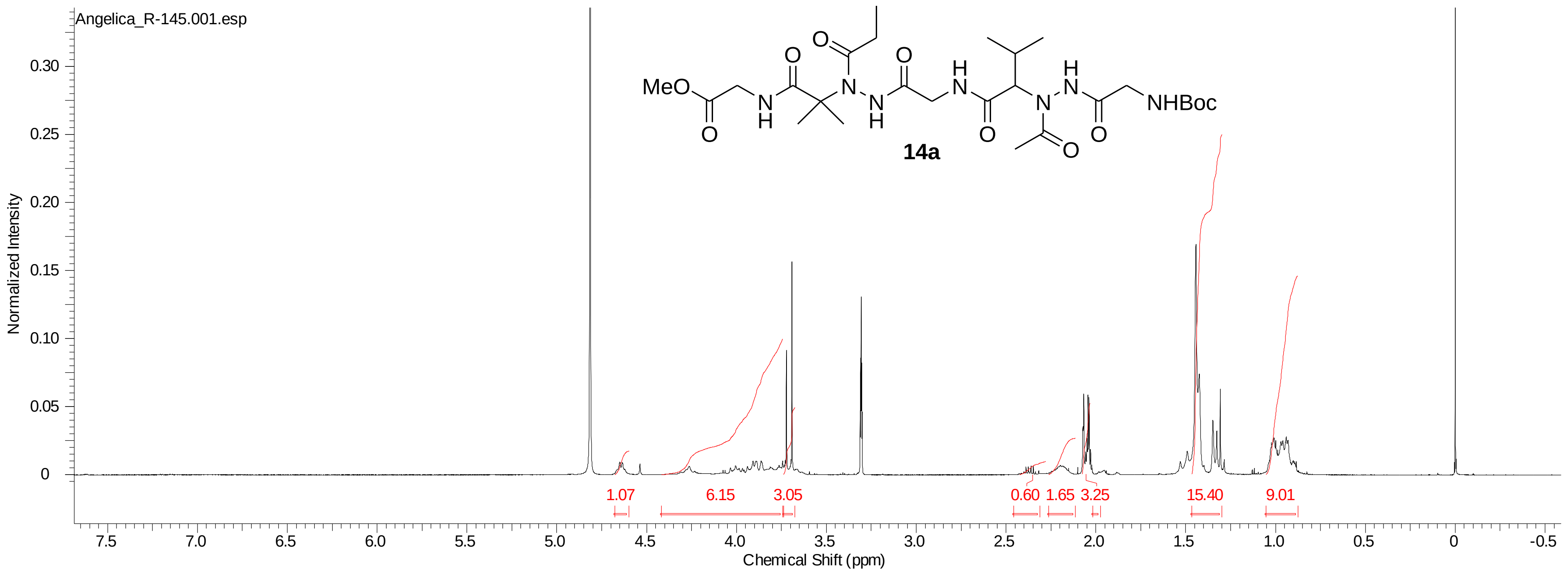

Figure S29: ^{1}H NMR (600 MHz, CD_3OD) spectrum of compound **14a**.

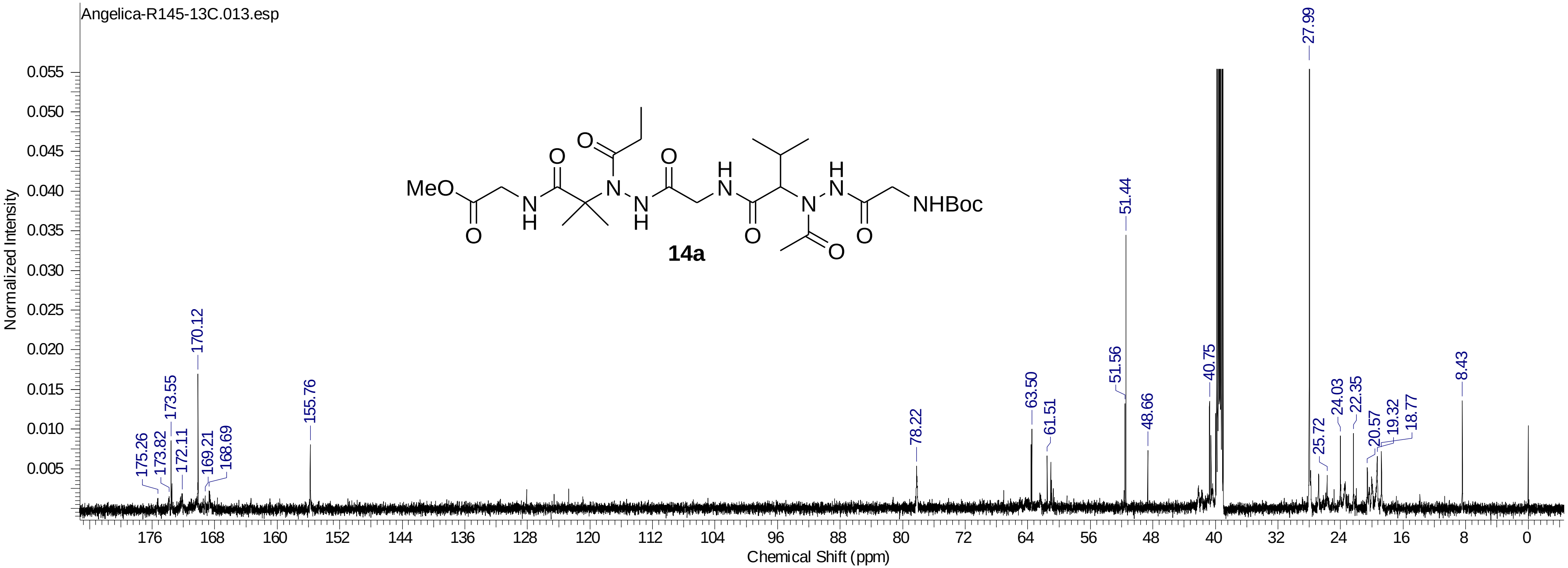

Figure S30: ^{13}C NMR (150 MHz, DMSO-d_6) spectrum of compound **14a**.

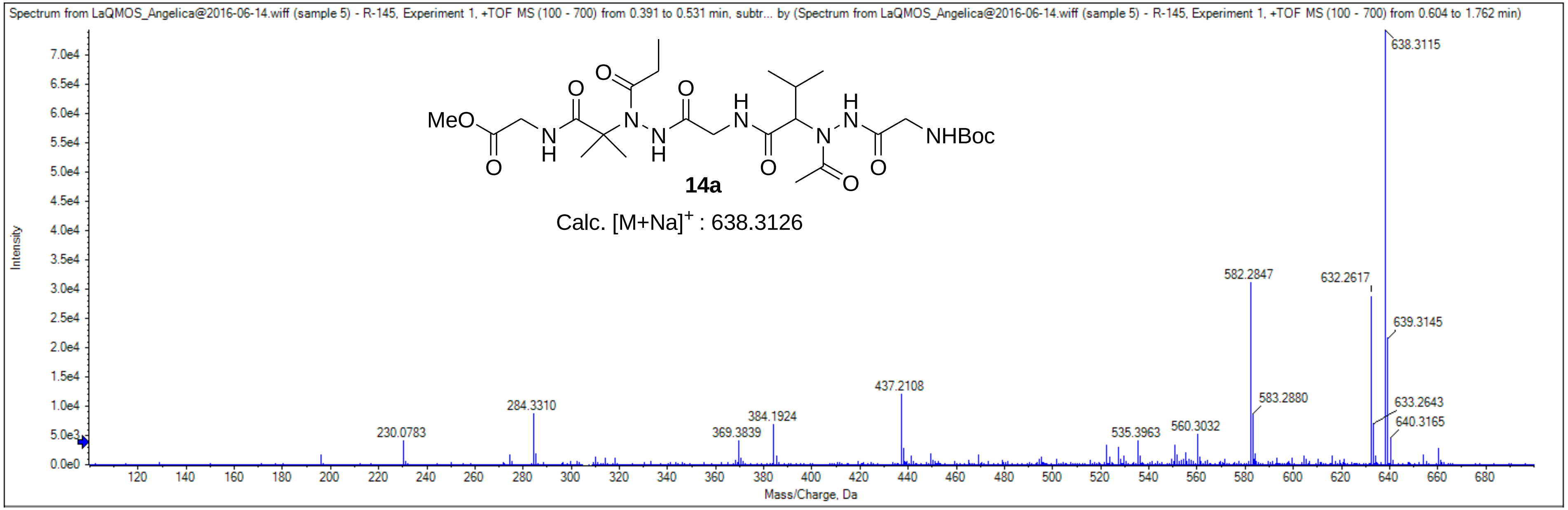

Figure S31: ESI-HRMS of compound **14a.**

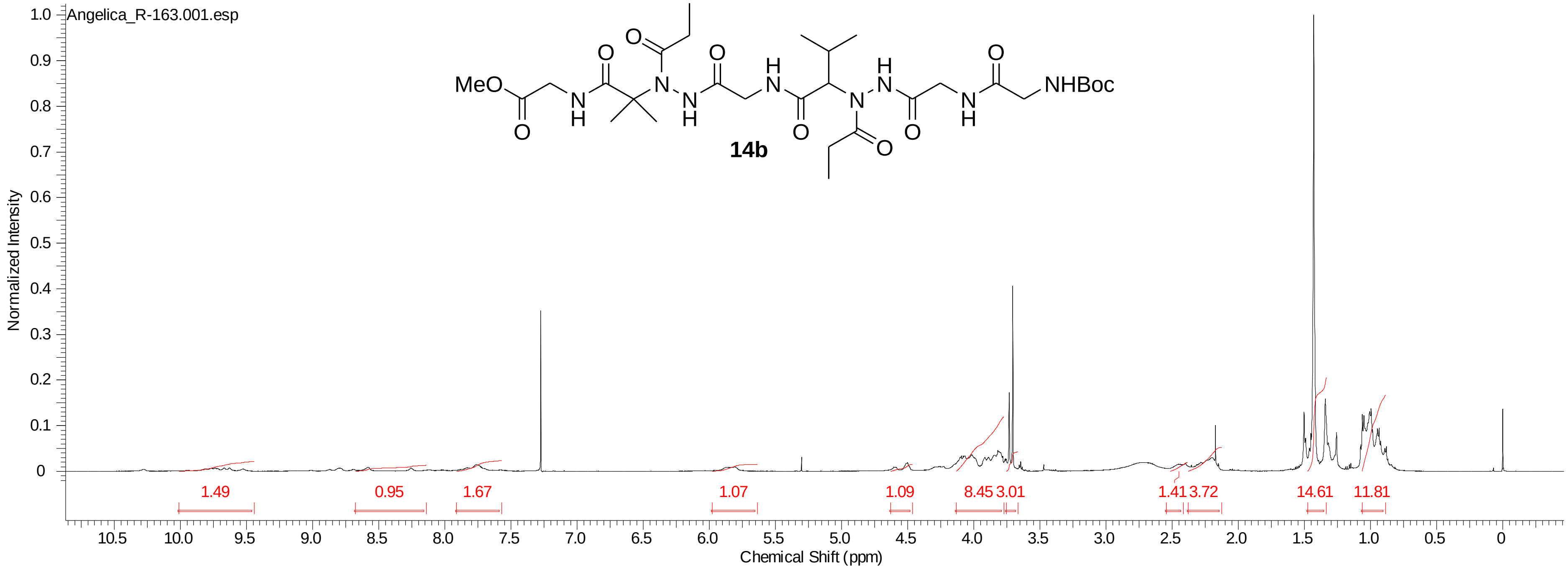

Figure S32: ^{1}H NMR (600 MHz, $CDCl_3$) spectrum of compound **14b**.

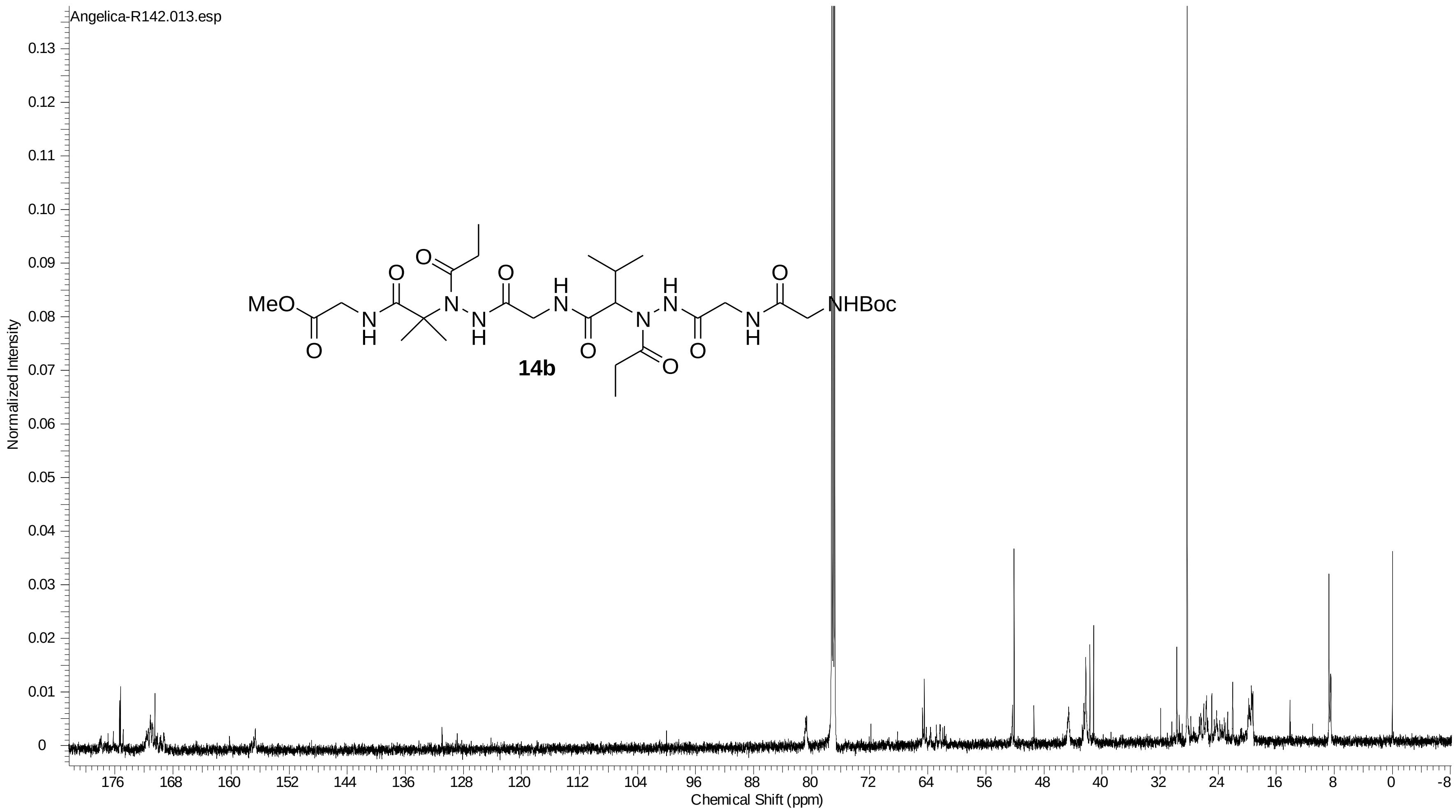

Figure S33: ^{13}C NMR (150 MHz, $CDCl_3$) spectrum of compound **14b**.

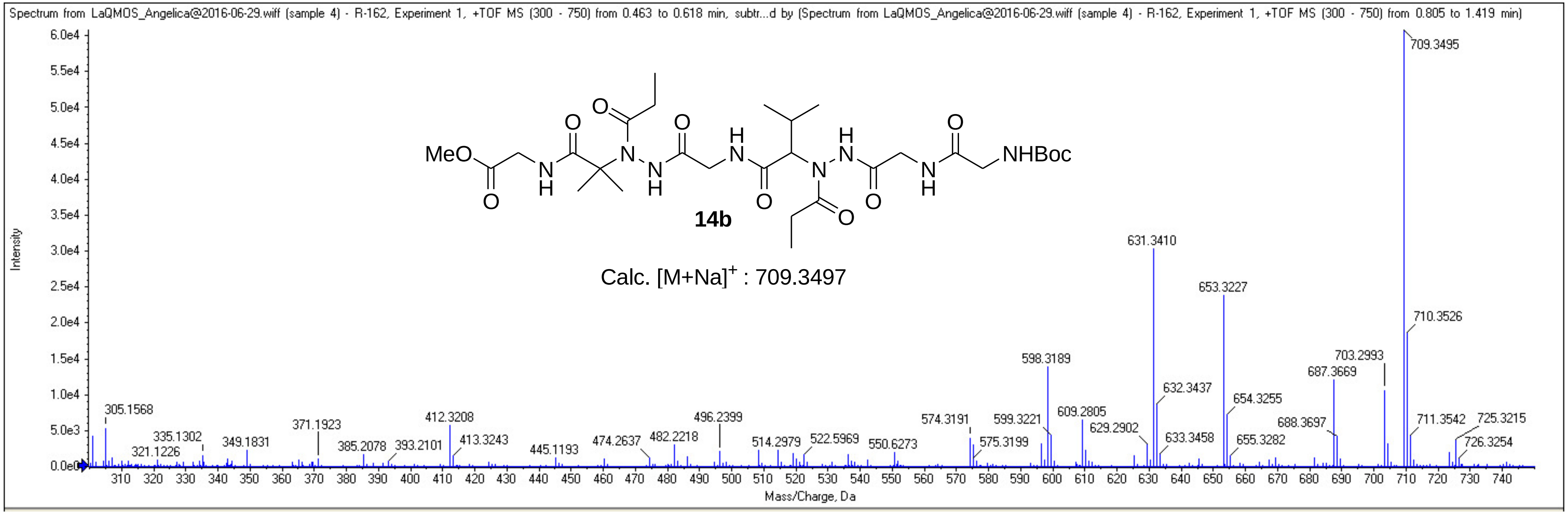

Figure S34: ESI-HRMS of compound **14b.**

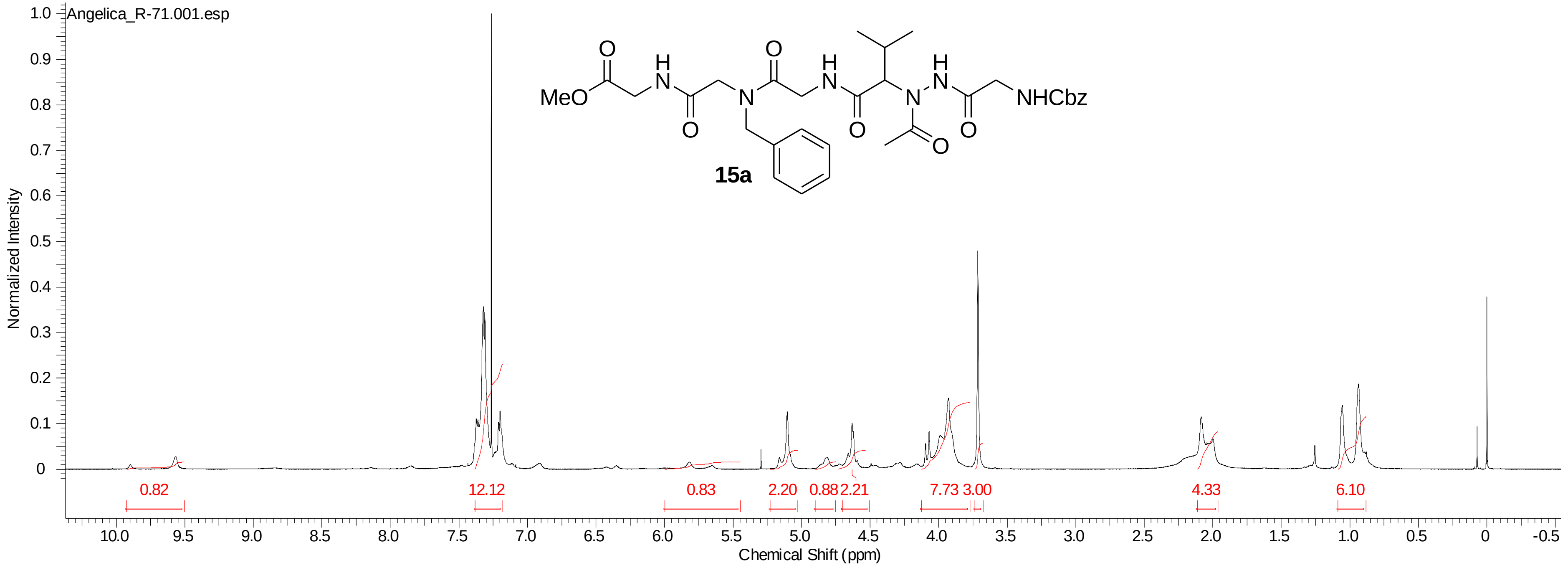

Figure S35: ^{1}H NMR (600 MHz, $CDCl_3$) spectrum of compound **15a**.

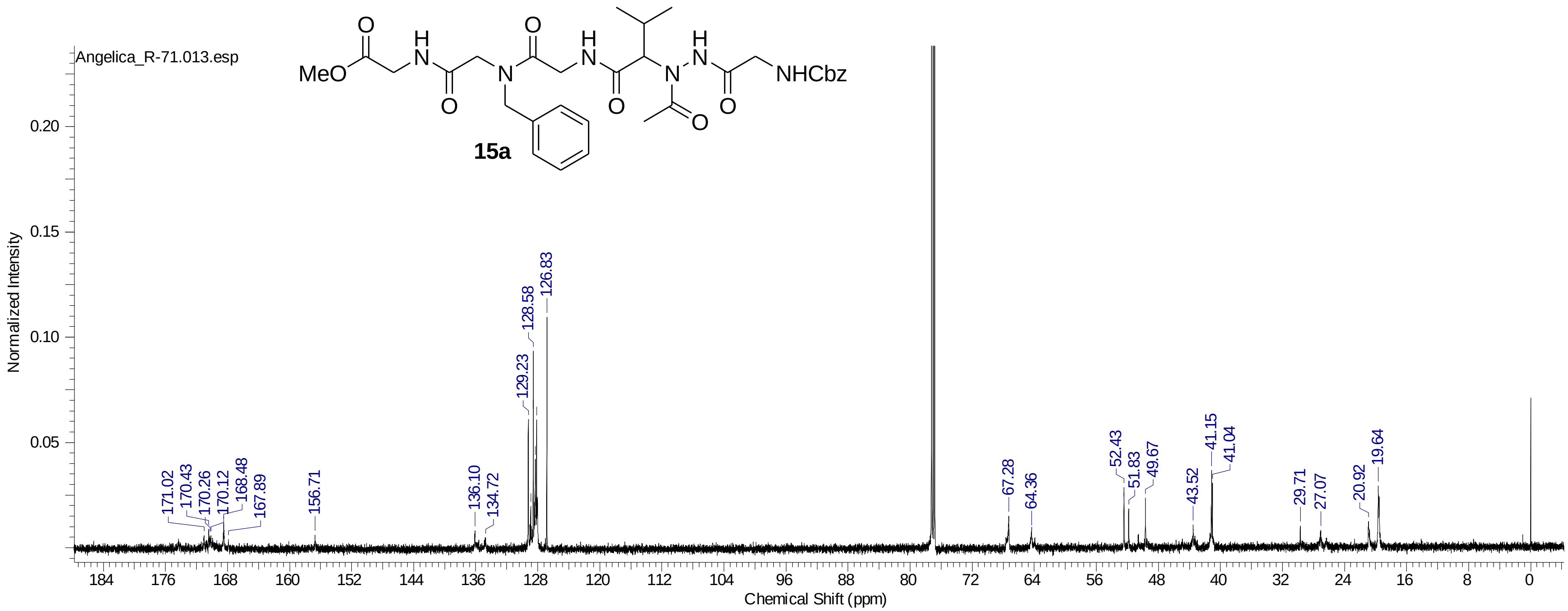

Figure S36: ^{13}C NMR (150 MHz, $CDCl_3$) spectrum of compound **15a**.

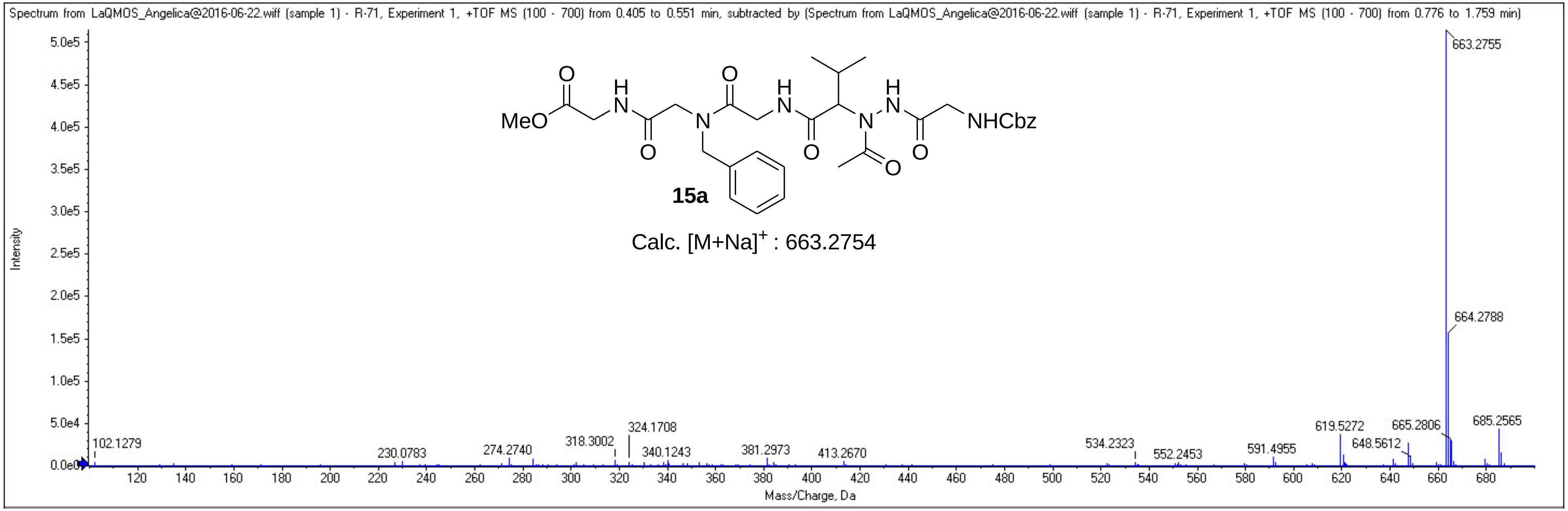

Figure S37: ESI-HRMS of compound **15a.**

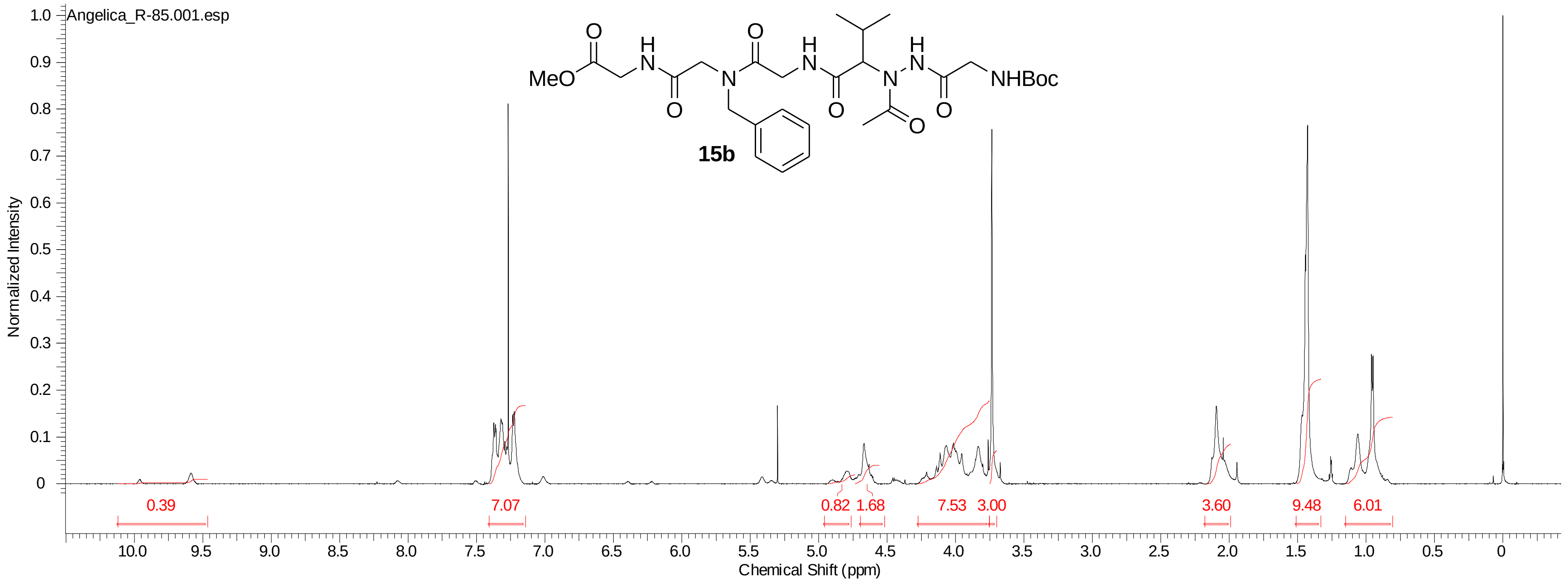

Figure S38: ^{1}H NMR (600 MHz, $CDCl_3$) spectrum of compound **15b**.

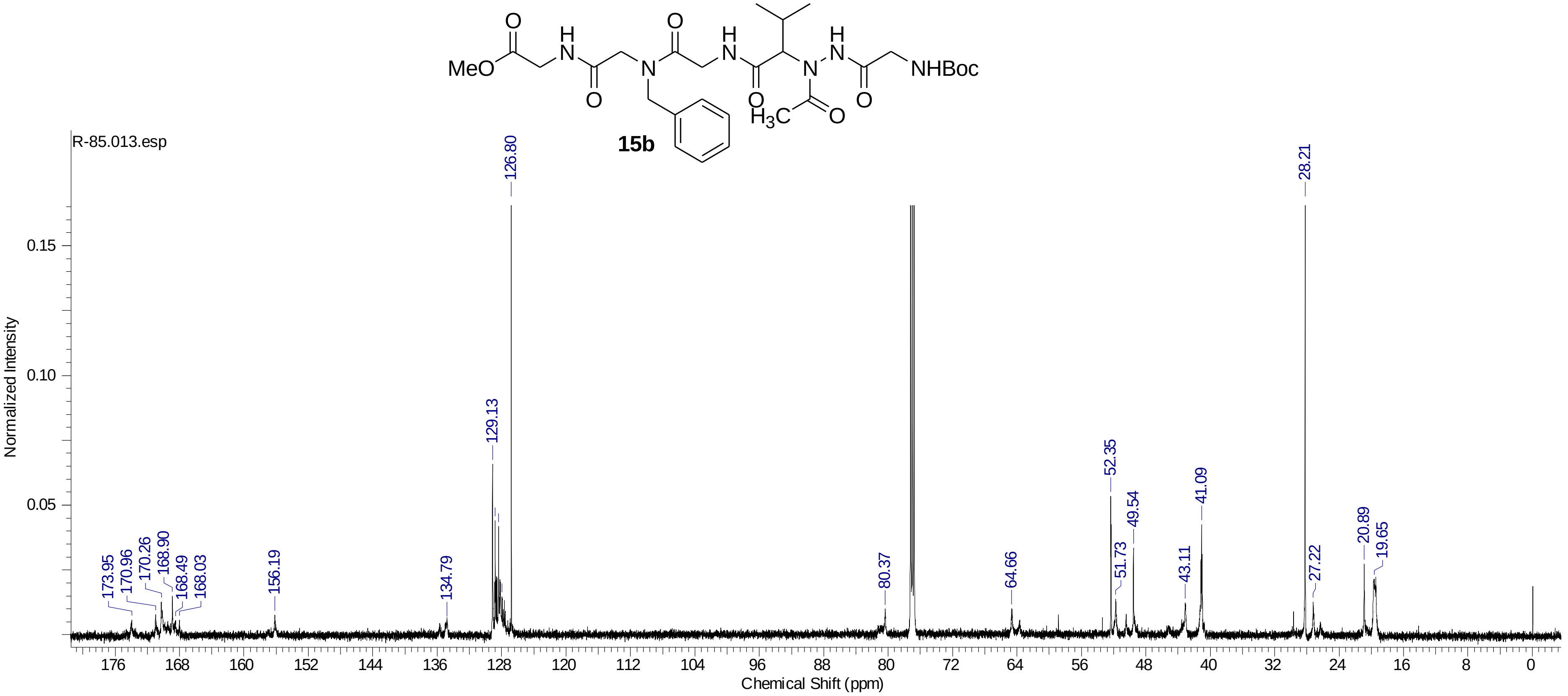

Figure S39: ^{13}C NMR (150 MHz, DMSO-d_6) spectrum of compound **15b**.

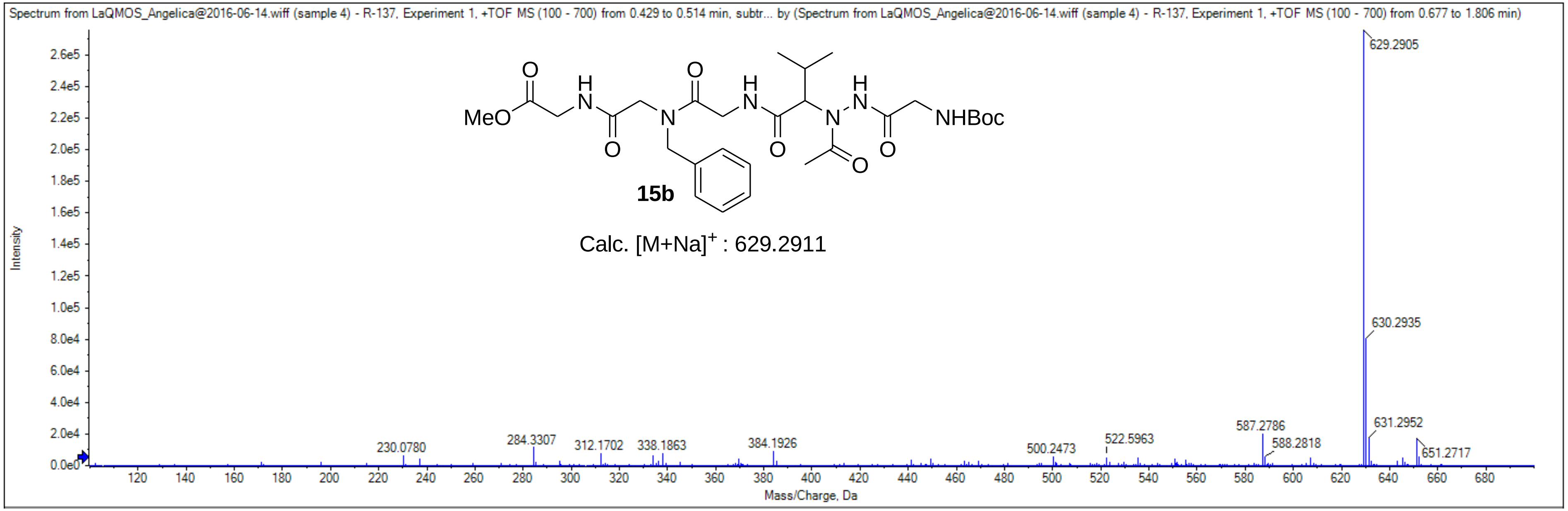

Figure S40: ESI-HRMS of compound **15b.**

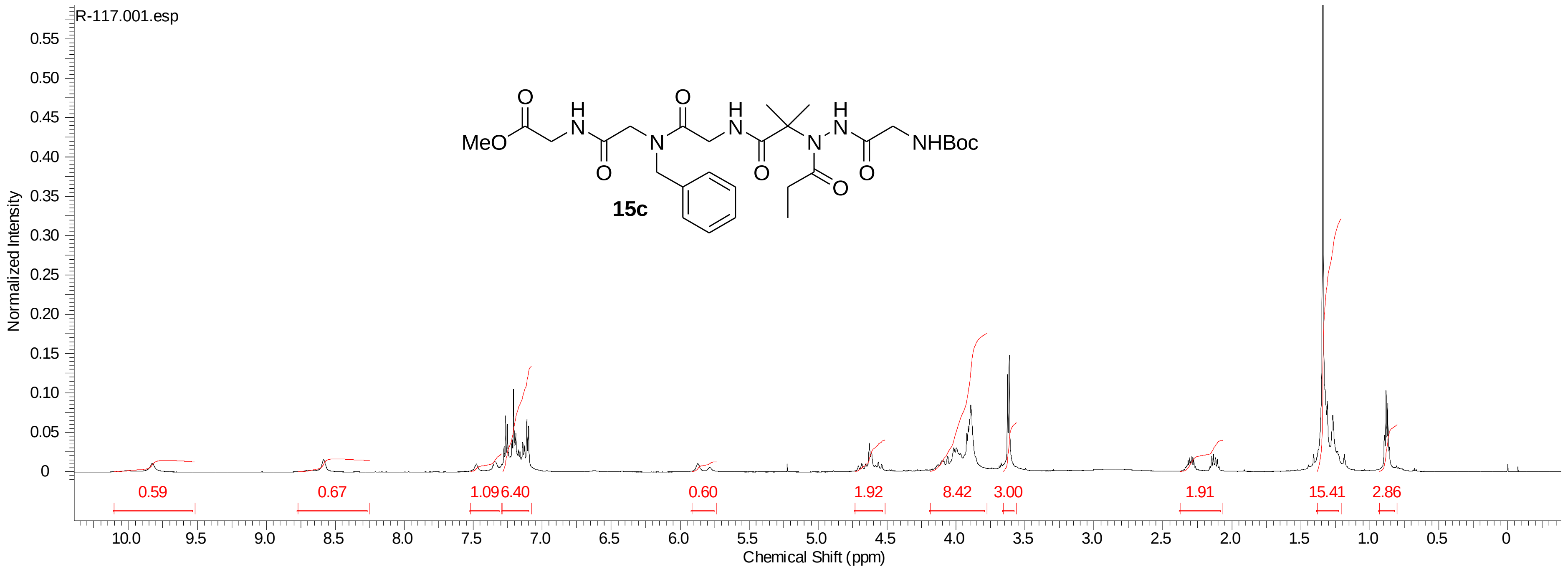

Figure S41: ^{1}H NMR (600 MHz, $CDCl_3$, presence of rotamers) spectrum of compound **15c**.

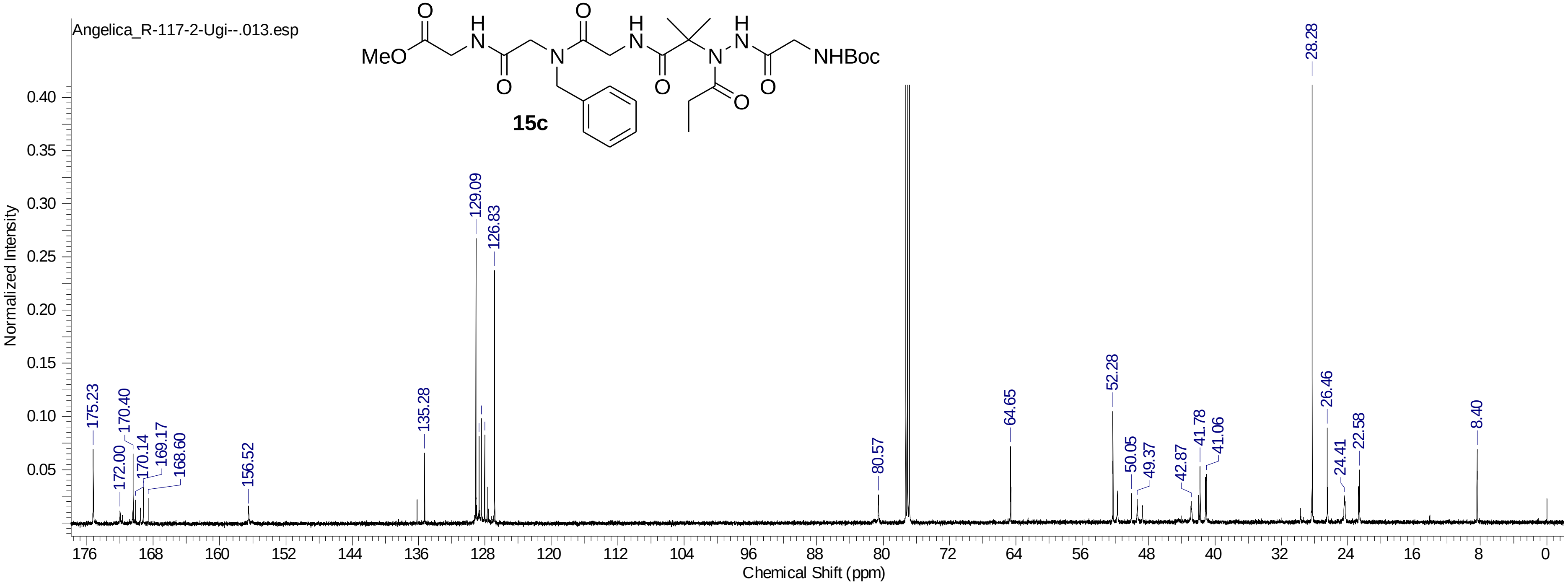

Figure S42: ^{13}C NMR (150 MHz, $CDCl_3$, presence of rotamers) spectrum of compound **15c**.

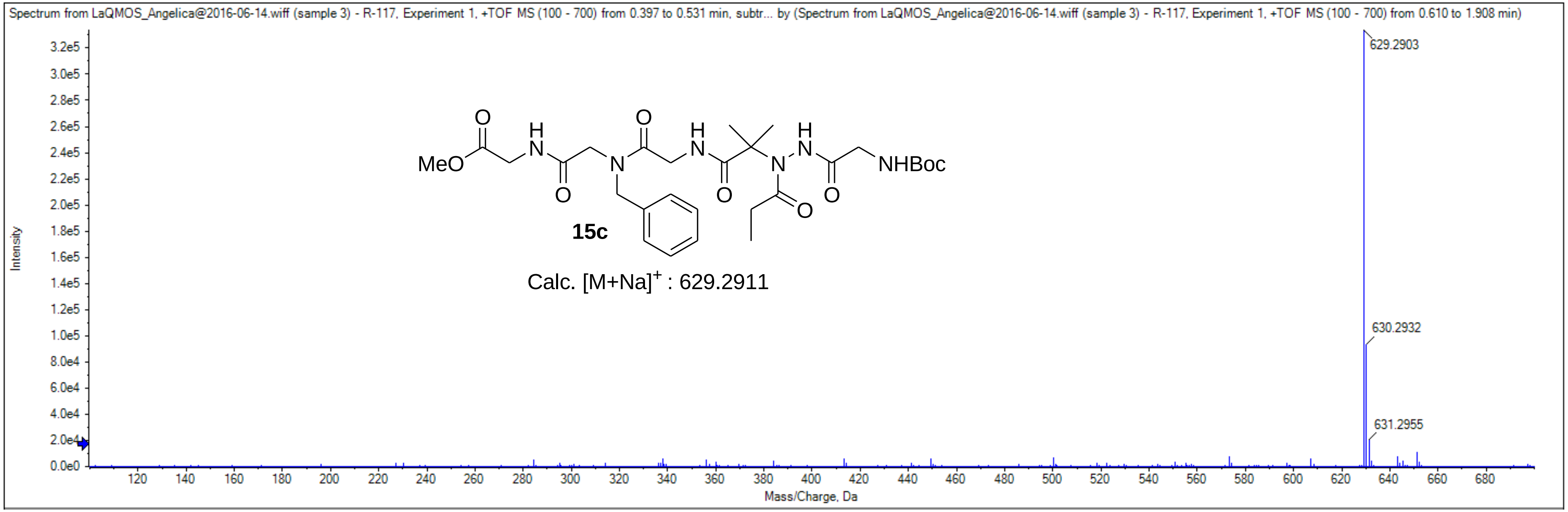

Figure S43: ESI-HRMS of compound **15c.**
